# Microfluidic engineering of pDNA nanogels in a coaxial flow reactor: process development, optimisation, scalability and *in vitro* performance

**DOI:** 10.1039/d5na00558b

**Published:** 2025-10-22

**Authors:** Suneha Patil, Zoe Whiteley, Esther Osarfo-Mensah, Arun Pankajakshan, Duncan Q. M. Craig, Stefan Guldin, Pratik Gurnani, Asterios Gavriilidis

**Affiliations:** a Department of Chemical Engineering, University College London Torrington Place London WC1E 7JE UK a.gavriilidis@ucl.ac.uk; b Department of Pharmaceutics, UCL School of Pharmacy, University College London 29-39 Brunswick Square London WC1N 1AX UK p.gurnani@ucl.ac.uk

## Abstract

Polymeric nanogels hold strong promise for gene delivery, but their production is often limited by poor scalability and inconsistent control over physicochemical properties. To address this challenge, we present a scalable microfluidic strategy for engineering carboxymethyl chitosan-grafted branched polyethyleneimine plasmid DNA nanogels (CMC-bPEI-pDNA NGs) using a coaxial flow reactor. This continuous flow platform enables precise control over nanogel formation, offering tunability in particle size, surface charge, and encapsulation efficiency. Through systematic process development and parametric optimisation – including investigations into hydrodynamics, mixing, reactor geometry, and effect of reagent concentrations – we designed a novel process achieving high-throughput, reproducible nanogel production suitable for *in vitro* gene delivery. Optimised formulations, produced in as little as 3 s residence time, exhibited excellent monodispersity (polydispersity index, PDI < 0.2), sub-200 nm particle size, and pDNA encapsulation efficiency exceeding 90%. Fluorescence microscopy-based transfection assays confirmed effective intracellular delivery with high green fluorescent protein (GFP) expression in HEK293T cells 72 h post-transfection. We successfully scaled the process 100-fold by extending the reactor length, while maintaining similar physicochemical properties and biological performance. Nanogels produced at high throughput (1.14 L h^−1^) maintained a high GFP expression, confirming functional gene delivery and process scalability. We identified critical process parameters governing nanogel properties and scalability, including minimum residence time for nanogel formation, optimal flow rate ratios, reagent feeds configuration and reactor design for large-scale implementation. This work establishes a robust and scalable microfluidic process for producing functional polymeric nanogel gene delivery vectors, demonstrating its feasibility for translation from laboratory to larger-scale manufacturing, thereby serving as a proof of concept for future industrial-scale gene therapy applications.

## Abstract 2

A scalable microfluidic process enables the production of functional polymeric nanogel gene delivery vectors, demonstrating feasibility for translation from lab to large-scale manufacturing.

## Graphical abstract

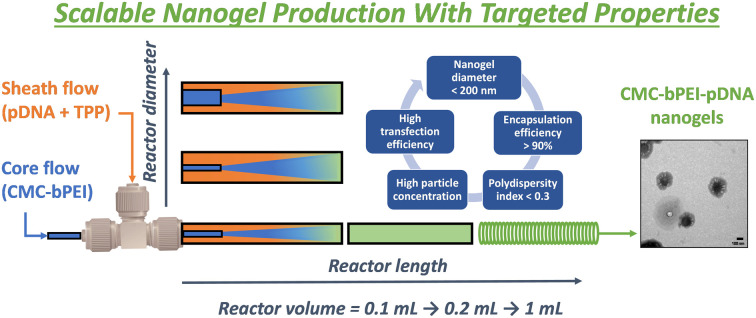

## 1 Introduction

Gene therapy and nucleic acid-based therapeutics aim to treat genetic diseases by targeting their root cause. Recent advances have enabled long-lasting and even curative outcomes for rare and complex diseases previously considered untreatable – offering a significant improvement over conventional treatments that target proteins rather than the underlying genetic defects.^1^ However, effective gene therapy technologies heavily rely on vectors to deliver genetic material across cell membranes as the payload is only functional inside the nucleus.^1^ While viral vectors are efficient, safety related risks associated with their use has driven the search for alternative non-viral vectors.^2^ Nanoparticle delivery systems, including lipid nanoparticles, polymeric, and inorganic/metallic nanoparticles, have been developed as promising non-viral platforms for nucleic acid delivery.^3^ Nanoparticles offer a high surface area, the ability to encapsulate genetic material, and tunable properties such as composition, size, and charge that can be engineered to overcome delivery barriers and address therapeutic challenges.^4^ However, to improve accessibility and enable widespread use, synthesis of non-viral vectors must be scalable while retaining their functionality. Among these, lipid nanoparticles are the first non-viral gene delivery systems to be clinically approved and scaled up for widespread use, enabling the delivery of billions of doses of COVID-19 mRNA vaccines: a significant milestone in both the fight against the pandemic and the advancement of non-viral gene-based therapies.^5^ Despite their success as vaccines, lipid nanoparticle formulation is complex, involving the self-assembly of multiple lipid precursors (typically 4) which require organic solvents for dissolution, followed by laborious post-processing steps to remove residual solvents and minimize toxicity.^6^ In comparison, polymeric nanogels are an emerging class of non-viral vectors that are typically produced *via* ionic gelation, a simple and rapid aqueous phase process wherein cross-linking occurs in the presence of oppositely charged ions. This method avoids the use of organic solvents and harsh conditions, offering advantages in biocompatibility and facilitating scale-up.^7^

Nanogels are three-dimensional (3D), nanosized hydrogels formed through chemical and/or physical cross-linking of polymer chains. They exhibit high-water content, biocompatibility with tissue and blood, ability to encapsulate and protect genetic material from enzymatic degradation^8,9^ and rapid responsiveness to microenvironmental factors (*e.g.* temperature, pH). These properties are tunable through modifications to their 3D structure,^10,11^ making them highly adaptable. Compared to other nanocarriers, nanogels offer broader compatibility with diverse cargos, including therapeutic and diagnostic agents, thereby enhancing their functionality. Their biodegradability can be tuned by incorporating biodegradable polymers as well as synthetic polymers with unstable linkages, and they can be engineered for selective responsiveness, and site-specific delivery.^12^ Key factors influencing nanogel performance include polymer type and concentration—typically represented by the N/P ratio (the ratio of moles of nitrogen in the polymer to phosphates in the biomolecule and cross-linker)—as well as nanogel size, surface charge, and encapsulation efficiency. For effective gene delivery, nanogels should ideally have a diameter of < 200 nm, a polydispersity index (PDI) < 0.3, encapsulation efficiency (EE) > 90%, and a high transfection efficiency. The synthesis method and process, particularly mixing efficiency, applied shear, and reactor hydrodynamics are critical in dictating these properties.^13^ Nanogel formation is highly sensitive to these factors, which significantly influence the resulting physicochemical properties, and consequently gene delivery performance. Despite extensive research on formulation development, most syntheses still relies on manual mixing of reagents,^14,15^ leading to irreproducibility, poor control over physicochemical properties and elevated production costs.^15^ Inefficient mixing in batch reactors often results in polydisperse particles and inconsistent product quality,^16–18^ posing significant barriers to scalable and reproducible manufacturing.^19^ Current iterative batch mixing at small scale often fails to replicate the dynamics and conditions encountered during large-scale manufacturing. Additional challenges, such as low transfection efficiency,^20^ complex formulation chemistries and batch variability^21–26^ hinder scalable production and limit clinical translation of nanogels for gene delivery.^21,22^ Despite these limitations, scalable and continuous nanogel manufacturing remains underexplored, underscoring the need for robust, efficient production methods to meet growing demand for non-viral vectors. Process optimisation is essential to establish a sustainable manufacturing workflow that ensures both the quality and efficacy of these vectors. In this study, we address this challenge by using a microfluidic coaxial flow reactor to develop a novel, scalable and fully optimised process for polymeric nanogel synthesis.

Microfluidic technologies offer precise control over fluid dynamics at the microscale, enabling fine-tuning of nanocarrier properties such as size and morphology. Devices such as the Y and T-mixers,^27^ staggered herringbone mixer,^28,29^ confined impinging jet reactor^30,31^ and the coaxial flow reactor have been employed to synthesize carriers for gene delivery *in vitro* and *in vivo*.^32^ The confined impinging jet reactor requires precise flow control to maintain equal momentum between opposing jets, and minor deviations can compromise mixing efficiency. Its design also demands accurate alignment of the impingement point, adding to the system's complexity and cost. Mixers such as the staggered herringbone mixers have been employed in commercial systems like the NanoAssemblr Benchtop (Precision Nanosystems).^28^ The staggered herringbone mixer was later scaled by integrating 128 parallel mixing channels, achieving nanoparticle production rates up to 18.4 L h^−1^ for mRNA and siRNA delivery.^29^ However, reliance on commercial production systems increases costs, creating a bottleneck for large-scale translation and limiting opportunities for innovative reactor designs. While large-scale production *via* microfluidics has been demonstrated,^29,33^ these efforts are specific to lipid nanoparticles, with little focus on polymeric nanogels.

To enable continuous flow process development and eventual scale-up, our initial focus was on the design and fabrication of a versatile continuous flow reactor. Reactor selection was based on our prior work demonstrating that the coaxial flow focusing reactor effectively produced chitosan-based carriers for protein^34^ and viral vector delivery.^35^ Flow focusing offers a significant advantage for nanomaterial production, enabling efficient mixing even under laminar flow conditions, making it particularly well-suited for gene delivery materials. Notably, clogging can be mitigated by ensuring nanoparticles are produced away from the reactor walls to avoid deposition upon wall contact.^36^ This contrasts with Y- and T-mixers, which, although simpler, often require high flowrates to ensure effective mixing and are prone to fouling in laminar flow operations.^37,38^ Flow-focusing has been previously used to tailor hyaluronic acid based nanogels *via* water–oil microemulsions.^39^ However, this process requires pressurisation to control droplet size and lengthy post processing to remove the organic phase (up to 2 days), thus posing scale-up challenges. In addition, the fabrication of microfluidic chips (*e.g.* high-throughput parallelised staggered herringbone mixer^29^ and other flow focusing devices^39–41^) often requires intricate design, complex lithographic techniques and the use of polydimethylsiloxane (PDMS) which suffers from absorption of small molecules, unstable surface wettability, deformation under high flowrates and pressure and poor chemical compatibility.^42,43^ Whether commercially sourced or fabricated in-house, the use of microfluidic chips remains time-consuming, costly, and dependent on specialised expertise. Collectively, these factors limit their scalability and practical use in continuous nanogel production. In contrast, the coaxial flow reactor used in this study is low-cost, modular and easy to assemble using readily available materials. Mixing in the coaxial flow reactor occurs under laminar flow conditions, ideal for processing shear-sensitive genetic materials.

In this work, we present a comprehensive study integrating continuous flow process development, *in vitro* transfection evaluation (*via* fluorescence-based transfection assays) and proof-of-concept scale-up for polymeric nanogels. Specifically, we focus on the continuous flow manufacturing of CMC-bPEI-pDNA nanogels *via* ionic gelation, using negatively charged plasmid DNA (pDNA), sodium tripolyphosphate (TPP) as the cross-linker and the cationic conjugated polymer (CMC-bPEI). The gene delivery performance of these nanogels is closely tied to their physicochemical characteristics, which are governed by parameters such as reagent concentrations (polymer, cross-linker, genetic material), reactor residence time, mixing efficiency, inlet flowrate ratios, and reagent feeds configuration. We systematically investigate the influence of these variables to establish a robust, scalable process, capable of producing nanogels with specific target properties (diameter < 200 nm, PDI < 0.3, encapsulation efficiency > 90%, and high transfection efficiency) at high throughputs.

**Fig. 1:**
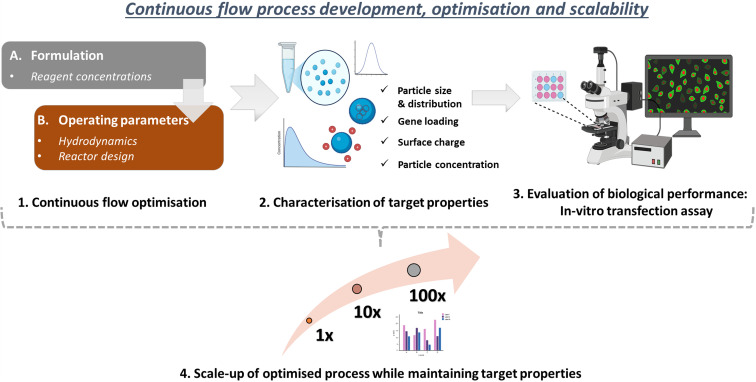
Flowchart illustrating the key steps in process development and scale-up of nanogels.

To our knowledge, this is the first study to report a 100-fold scale-up (0.01 L h^−1^ to 1.14 L h^−1^) in continuous polymeric nanogel production, coupled with a systematic investigation of how individual process variables affect their properties and gene delivery performance. Current literature provides limited insight into the scale-up pathway for polymeric gene delivery systems and how continuous flow parameters influence nanogel functionality. This lack of scalability data poses a major barrier to advancing nanogels towards practical applications. Our work addresses these gaps by systematically evaluating key variables in flow-based nanogel manufacturing and identifying critical considerations for translating their production to higher throughputs. Unlike previous coaxial flow reactor based studies emphasising formulation, this study prioritises the development and optimisation of a continuous flow process to achieve high-throughput, scalable nanogel production. Moreover, we confirm the gene delivery potential of the resulting nanogels through *in vitro* transfection studies. As illustrated in Fig. 1, we begin by optimising nanogel formulation in continuous flow (**Section 3.1**), followed by optimisation of the reactor operating conditions (**Section 3.2**) to maintain target properties and transfection efficiency. Finally, we establish a scalable pathway for high-throughput nanogel synthesis (**Section 3.3**) while maintaining these properties.

## 2 Methodology

### 2.1 Reactor design and experimental setup

**Fig. 2:**
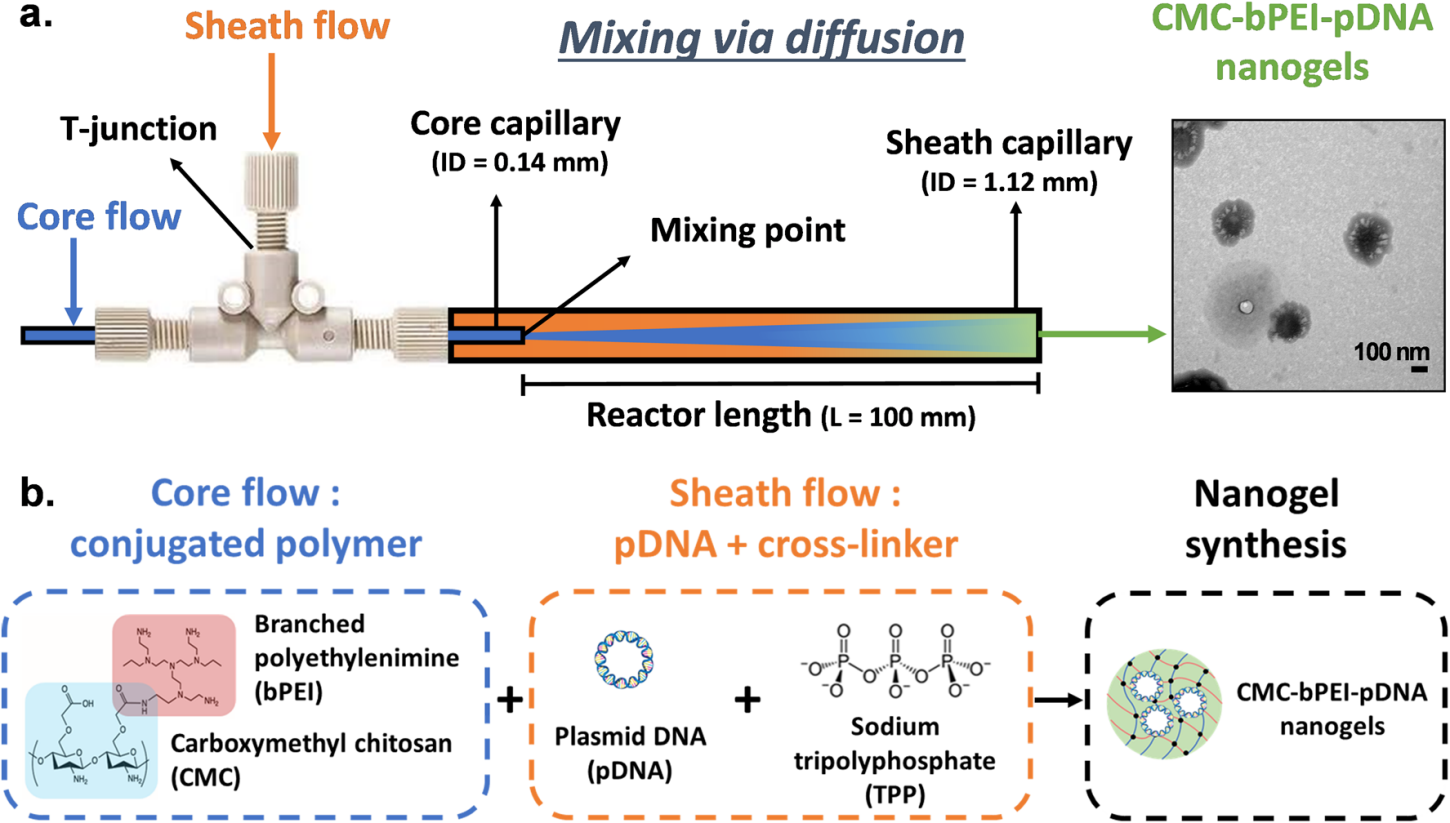
(a) Schematic of the coaxial flow reactor (CFR V3) in its standard configuration = PIC, where the polymer forms the core stream and the pDNA–TPP mixture forms the sheath, and (b) reagents used in the synthesis.

The coaxial flow reactor features a small diameter inner capillary nested within a larger outer capillary, generating a core flow surrounded by a sheath flow (Fig. 2(a)). Mixing starts at the outlet of the inner capillary (where the core and sheath streams meet), with the reaction proceeding along the channel length. To build the coaxial flow reactor, a core capillary (inner tube) and a sheath capillary (outer tube) (Glass, VWR, UK) were assembled in a coaxial, tube-in-tube configuration using a PEEK T-junction (0.050″ bore, VWR, UK). The T-junction was custom drilled: one port was modified to match the outer diameter (OD) of the core capillary, ensuring a snug fit and the opposite port was adjusted to fit the inner diameter (ID) of the sheath capillary. This configuration positioned the core stream centrally within the coaxial flow reactor. The reactor assembly is shown in Fig. S1, SI. Core capillaries of ID 0.59 and 0.14 mm and sheath capillaries of ID 1.6 and 1.12 mm were used to make different reactors as indicated in Table 1. The reactor length (see **Section 3.3.1**) for CFR V4 was increased by joining another glass capillary (ID 1.12 mm) *via* a PEEK union (0.045″ bore). The reactor length for CFR V5 was increased by further connecting 0.8 m PTFE tubing (ID 1.14 mm) to the reactor assembly V4 through a second PEEK union (0.045″ bore). The additional PTFE tubing was coiled to obtain a radius of curvature of 50 mm. Two 10 mL glass syringes (SGE Analytical Science) were filled with the reagent solutions and loaded on to syringe pumps (KDS Legato 200) to deliver the solutions to the reactor. All the tube connections (PTFE tubing, VWR, UK) from syringes to the reactor were 1 mm ID (1/16″ OD). The product was collected in a 5 mL glass vial at the outlet of the reactor.

**Table 1:** Details of coaxial flow reactor designs studied in this work

Dimensions	Coaxial flow reactor, CFR				
	V1	V2	V3	V4	V5
Sheath capillary ID (mm)	1.6	1.6	1.12	1.12	1.12
Sheath capillary OD (mm)	3	3	1.5	1.5	1.5
Core capillary ID (mm)	0.59	0.14	0.14	0.14	0.14
Core capillary OD (mm)	0.82	0.6	0.6	0.6	0.6
Reactor length, *L* (mm) (distance between mixing point and reactor outlet)	100	100	100	200	1000

### 2.2 Flow synthesis of CMC-bPEI-pDNA nanogels

Nanogels were synthesised in the coaxial flow reactor in a temperature-controlled laboratory (22 ± 2 °C). A conjugated polymer – branched polyethyleneimine, bPEI grafted with carboxymethyl chitosan, CMC – was used, as CMC-bPEI has been reported to enhance biocompatibility, transfection and cell viability.^44–47^ Nanogels were formed by cross-linking the conjugated polymer (CMC-bPEI) and pDNA using sodium tripolyphosphate (TPP, Sigma Aldrich) as the cross-linker (Fig. 2(b)). For details on polymer conjugation procedure and plasmid DNA extraction, please refer to Section S1(b and c) in the SI. Reagent solution A – conjugated polymer, and solution B – a mixture of plasmid DNA and TPP, were prepared in nuclease free water (NFW, Severn Biotech Ltd). Using the two syringe pumps, solutions A and B were pumped simultaneously in the coaxial flow reactor. Mixing was initiated at the designated mixing point, located at the outlet of the core capillary, and the cross-linking reaction proceeded throughout the length of the reactor. The feeds configuration PIC (polymer in core stream and pDNA-TPP in the sheath stream) was used unless otherwise stated. The initially formed nanogels were discarded for approximately three residence times to allow the system to reach a steady state. Subsequently, samples were collected and stored at 4 °C for further analysis. All experiments (except those to evaluate the effect of reactor design on nanogels) were performed in CFR V3 (see Table 1). Details of reactor design and operation parameters for the latter are mentioned wherever applicable. For investigations on the effect of individual reagents on nanogel formation (**Section 3.1**) one parameter at a time: polymer, TPP cross-linker and pDNA, was varied, and Table 2 lists the final concentrations in the reactor (after mixing) for these experiments.

**Table 2:** Details of final concentrations (after mixing) used in flow optimisation of nanogel formulation. *P*_pDNA_ and *P*_TPP_ are molar concentration of phosphates in pDNA and TPP respectively, and *N* is the molar concentration of nitrogen in the polymer

Influence of	pDNA conc.		TPP conc.		Phosphate conc.	N/P ratio	CMC-bPEI conc.	
	(µg mL^−1^)	*P*_pDNA_ (µM)	(% W/V)	*P*_TPP_ (µM)	*P* = *P*_pDNA_+*P*_TPP_ (µM)	(—)	*N* (µM)	(% W/V)
N/P ratio (polymer conc.)	25	75.76	0.0095	778	854	0.2	171	0.0010
	25	75.76	0.0095	778	854	0.8	683	0.0039
	25	75.76	0.0095	778	854	1	854	0.0048
	25	75.76	0.0095	778	854	2	1707	0.0097
	25	75.76	0.0095	778	854	3	2561	0.0145
	25	75.76	0.0095	778	854	4	3414	0.0193
	25	75.76	0.0095	778	854	5	4268	0.0242
	25	75.76	0.0095	778	854	12	10 242	0.0580
	25	75.76	0.0095	778	854	30	25 606	0.1449

TPP conc. (cross-linker)	25	75.76	0.0095 (1×)	778	854	3	2561	0.0145
	25	75.76	0.0047 (0.5×)	389	465	3	1394	0.0079
	25	75.76	0.019 (2×)	1556	1631	3	4894	0.0277
	25	75.76	0.047 (5×)	3889	3965	3	11 894	0.0673
	25	75.76	0.095 (10×)	7778	7854	3	23 561	0.1334

pDNA conc. (gene loading)	12.5	37.88	0.0100	816	854	3	2561	0.0145
	25	75.76	0.0095	778	854	3	2561	0.0145
	50	151.52	0.0086	702	854	3	2561	0.0145
	75	227.27	0.0077	626	854	3	2561	0.0145
	100	303.03	0.0067	551	854	3	2561	0.0145

### 2.3 Transfection of nanogel formulations

HEK293T cells were seeded at a density of 0.12 × 10^6^ cells per well in 1 mL of growth media in 12-well cell culture-treated plates. Once the cells reached 75–85% confluency, they were transfected with nanogel formulations, a positive control (Lipofectamine 3000, Invitrogen, Loughborough, UK), and a negative control (pDNA alone in the absence of a vector). For additional details on cell preparation and control experiments, refer to Section S1(d) in the SI. Nanogel formulations prepared at varying N/P ratios, TPP concentrations, pDNA concentrations, residence times, flowrate ratios and reactor volumes were used for transfection. Cells were transfected with a total pDNA content of 0.5, 1, and 1.5 µg per well. For formulations where polymer solution was used as the sheath stream, additional wells of total pDNA content − 2 and 2.5 µg were also tested.

To investigate the effect of pDNA concentration in the formulation, nanogels were prepared at final pDNA concentrations of 12.5 µg mL^−1^, 25 µg mL^−1^, 50 µg mL^−1^, 75 µg mL^−1^, and 100 µg mL^−1^. For transfections, a fixed pDNA amount of 1.5 µg was added to each well, corresponding to formulation volumes of 120 µL, 60 µL, 30 µL, 20 µL, and 15 µL, respectively. Formulation refers to the nanogel dispersion collected at the reactor outlet. Prior to transfection, the formulation volumes were adjusted to a final volume of 200 µL with Opti-MEM to ensure consistent well volumes. For further details on the formulation volumes used for 0.5, 1, 2, and 2.5 µg of total pDNA content per well, refer to Table S1 in the SI. Following transfection, cells were incubated at 37 °C in a 5% CO_2_ atmosphere and subsequently assessed for green fluorescent protein (GFP) expression *via* fluorescence microscopy.

### 2.4 Analysis and characterisation

#### 2.4.1 Nanogel physicochemical characterisation

The particle size, polydispersity index (PDI) and zeta potential (ZP) of nanogels were determined using dynamic light scattering (DLS). The DelsaMax PRO light scattering analyser (B23930AA, Beckmann Coulter) equipped with a 50 mW diode pumped solid state (DPSS) single-longitudinal-mode laser was used for particle size and PDI measurements. 30 µL freshly prepared nanogel suspensions were diluted in 1 mL nuclease free water (NFW) and placed in the analyser in disposable PMMA (poly(methyl methacrylate)) cells. All measurements were carried out in triplicate at 25 °C. For measurement of zeta potential, undiluted nanogel suspensions were loaded in folded capillary zeta cells (DTS1070, Malvern Panalytical, UK) and placed in a Zetasizer Ultra (Malvern Panalytical, UK) for triplicate measurements at 25 °C. Measurement data from three independent experiments were averaged out and expressed as mean size ± standard deviation. Particle concentrations were measured using nanoparticle tracking analysis (NTA) and their measurement procedure is available in methods Section S1(f) in the SI.

#### 2.4.2 Measurement of pDNA concentration and encapsulation efficiency (EE)

The concentration of pDNA obtained from maxiprep was measured using a Nanodrop 2000 UV-Vis spectrophotometer (Thermo Fisher Scientific, US). 1 µL sample of Tris-EDTA (TE) buffer was placed on the sample holder for a blank measurement. Following this, 1 µL of isolated pDNA was measured and the concentration in ng µL^−1^ was obtained. The pDNA was then stored at −20 °C. To measure the encapsulation efficiency of nanogels, 2 mL of nanogel formulation was centrifuged at 14 000×*g* for 30 min at room temperature. The supernatant was then measured for free pDNA in the Nanodrop spectrophotometer, and the encapsulation efficiency was calculated using eqn (1).1
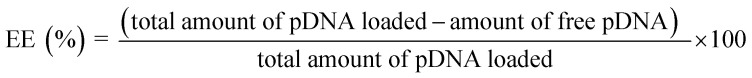


#### 2.4.3 Transmission electron microscopy (TEM)

Undiluted liquid samples for TEM were dropped with a Pasteur pipette onto a carbon/formvar coated copper grid. After 15 s, excess sample was blotted off with filter paper. Then a drop of 2% phosphotungstic acid was added and blotted after 15 s. The grid was placed into a specimen holder and inserted into a Phillips/FEI CM 120 BioTwin TEM for imaging at 120 kV.

#### 2.4.4 Assessment of green fluorescent protein (GFP) expression *via* fluorescence microscopy

Transfected cells were evaluated for GFP expression 72 h post-transfection using an EVOS FL fluorescence microscope (Thermo Fisher Scientific, Loughborough, UK). Multiple images were captured across the entire well to ensure full spatial representation of the well. Representative images reflecting the overall transfection efficiency observed within the well are presented.

Fluorescence microscopy images were analysed to assess GFP expression by detecting and quantifying fluorescent blobs (cells) using a custom Python script. Multiple images across three transfections were analysed to quantify GFP expression based on fluorescent blob count. The images were converted to grayscale, and fluorescent blobs were detected using the Laplacian of Gaussian (LoG) method. Quantitative results, including the fluorescent blob count were used for further statistical analysis to assess GFP expression levels and therefore transfection efficiency. For additional details on the quantitative assessment of the microscopy images, refer to Section S1(e) in the SI.

#### 2.4.5 Statistical analysis

Statistical data analysis was performed using GraphPad Prism 9.1.0 (GraphPad Inc., USA). One-way analysis of variance (ANOVA) was used to evaluate the effect of the independent variable (operation parameter, *viz.* N/P ratio) on more than two groups of the dependent variable (measurement data, *viz.* particle sizes at various N/P ratios). Two-way ANOVA was employed to determine the effect of two independent variables (*viz.* throughput and reactor design) and their potential interaction, on the dependent variable (measurement data, *viz.* particle size). *Post hoc* analysis included Tukey's multiple comparisons test, Dunnett's test, and Fisher's Least Significant Difference (LSD) test, where applicable. *P*-value < 0.05 was considered statistically significant.

## 3 Results and discussion

### 3.1 Influence of reagent concentrations

We first investigated the effect of each reagent (polymer, cross-linker and genetic material) on nanogel formation by varying one parameter at a time (Table 2). Optimisation was performed in coaxial flow reactor configuration 3 (CFR V3) due to its smallest working volume (0.1 mL) and therefore minimal reagent consumption. Operation parameters were fixed at feeds configuration PIC, flowrate ratio (FRR = ratio of volumetric flowrate of the core stream to the sheath stream) of 0.1 and a residence time of 31 s.

#### 3.1.1 Effect of nitrogen to phosphate (N/P) ratio

The N/P ratio is an important factor in polyelectrolyte complexation as this governs the relative charge ratio between the positively charged polymer and negatively charged nucleic acid. Hence the N/P ratio is known to impact particle size, surface charge and therefore transfection efficiency of these vectors.^48^ Here we varied the N/P ratio from 0.2 to 30, by varying the polymer concentration in the core stream. The total concentration of phosphates (supplied by pDNA and TPP) in the sheath stream was kept constant at 854 µM, and the final concentration of pDNA loaded in the formulations was 25 µg mL^−1^.

**Fig. 3:**
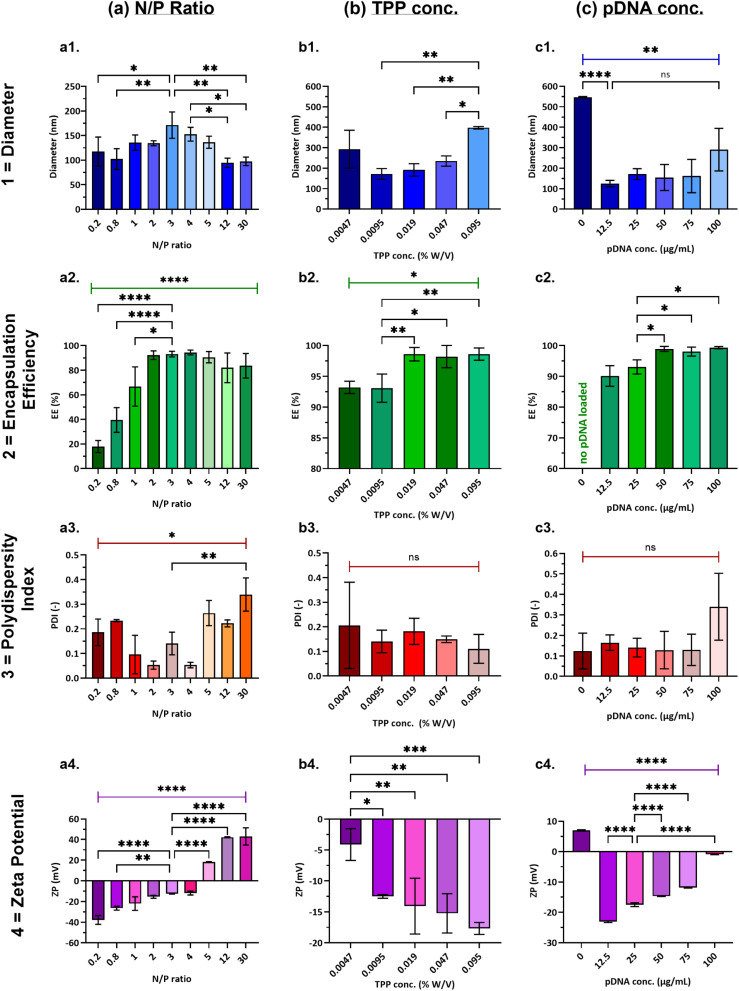
Physicochemical properties of nanogels produced with (a) increasing polymer concentration, N/P ratio (TPP = 0.0095% W/V, pDNA = 25 µg mL^−1^); (b) increasing TPP conc. (N/P = 3, pDNA = 25 µg mL^−1^) and (c) increasing pDNA conc. (N/P = 3, total phosphate conc. ∼854 µM). The measured physicochemical properties were 1 = nanogel size, (diameter); 2 = encapsulation efficiency, (EE); 3 = polydispersity index, (PDI); 4 = zeta potential, (ZP). Reactor operating conditions were fixed at – feeds configuration PIC, FRR = 0.1, RT = 31 s. A one-way ANOVA study and Tukey's multiple comparisons test were performed to assess the effect of reagent concentrations on each of the dependent variables – diameter, EE, PDI and ZP, separately. Plots illustrate mean ± standard deviation and the results from Tukey's HSD, comparing the means of each reagent concentration with one another for each dependent variable. *P < 0.05, **P < 0.01, ***P < 0.001, ****P < 0.0001, ns = not significant (*P* > 0.05). For further details on reagent concentrations, refer to Table 2.

**Fig. 4:**
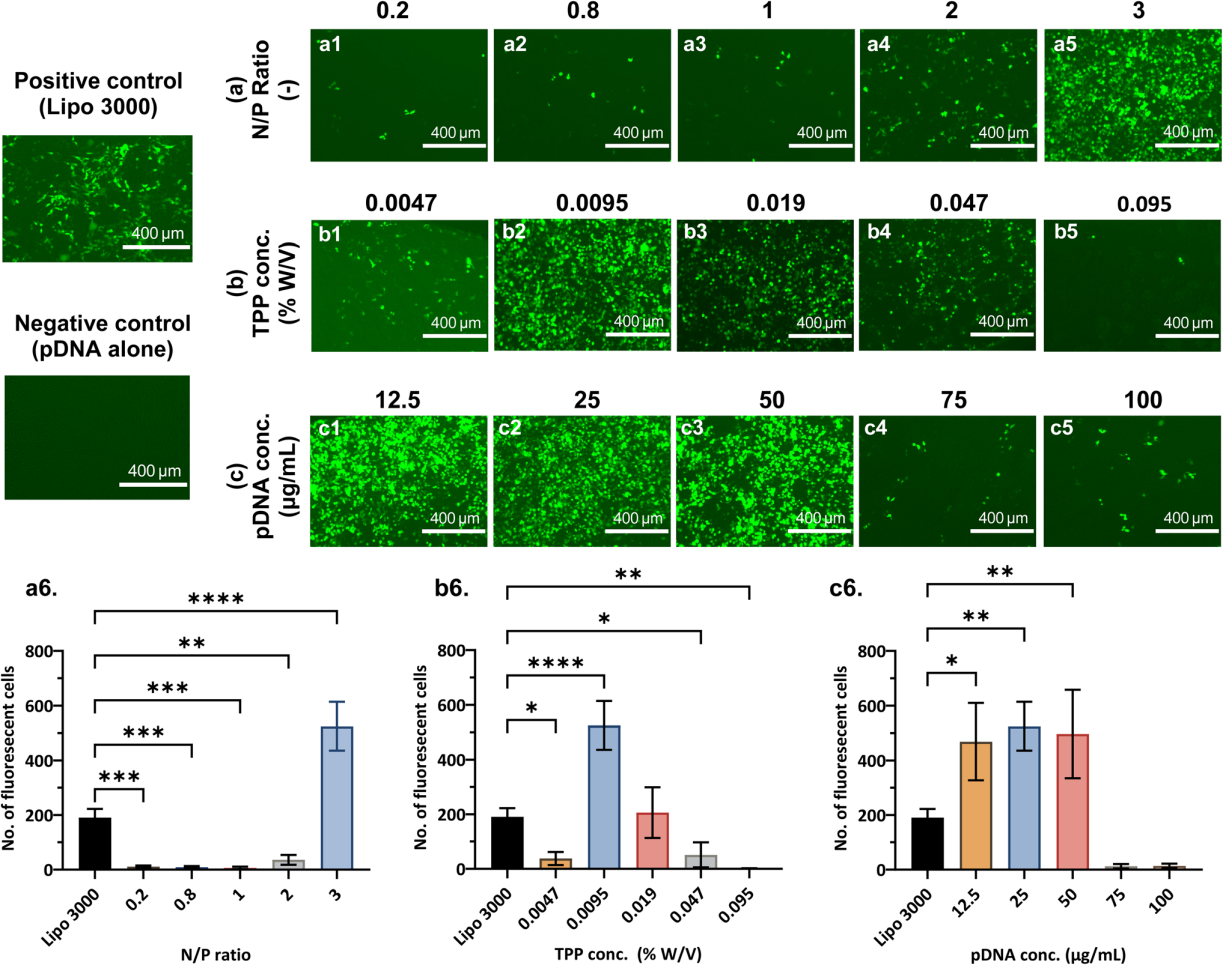
GFP expression in HEK293T cells at 72 h post-transfection. (Top) Fluorescence microscopy images (read left to right) of positive control – Lipofectamine 3000, negative control – pDNA alone without a vector, and (a1–a5) nanogels synthesised with N/P ratios 0.2, 0.8, 1, 2, 3; (b1–b5) nanogels synthesised with TPP concentrations 0.0047% (0.5×), 0.0095% (1×), 0.019% (2×), 0.047% (5×), 0.095% (10×); (c1–c5) nanogels loaded with pDNA concentrations 12.5, 25, 50, 75 & 100 µg mL^−1^. (Bottom) Quantitative GFP assessment – plots show the number of fluorescent cells detected for the positive control and nanogels produced at varying reagent concentrations: (a6) N/P ratio, (b6) TPP concentration, and (c6) pDNA concentration. Plots illustrate mean ± standard deviation and the results from Dunnett's *post hoc* tests, comparing the means of each reagent concentration with the control – Lipofectamine 3000. All nanogel formulations were synthesised with reactor operating parameters: FRR = 0.1 and RT = 31 s. The pDNA content per well for controls and nanogel formulations was standardised at 1.5 µg per well during transfection. For detailed reagent concentrations, refer to Table 2. *P* < 0.05, **P < 0.01, ***P < 0.001, ****P < 0.0001.

Encouragingly all particle sizes obtained for all N/P ratios were below 200 nm, a rough threshold for improved uptake in cells (Fig. 3(a1)). A statistically significant increase in nanogel diameter was seen when increasing N/P from 0.2 to 3 (*P < 0.05), after which it decreased to as low as 98 ± 9 nm for the highest polymer concentration of N/P = 30 (**P < 0.01). The pDNA encapsulation efficiency increased steadily from < 20% to > 90% (Fig. 3(a2)) up to N/P = 2 and remained almost quantitative until N/P = 30, due to the presence of more cationic polymer (****P < 0.0001). Furthermore, nanogels remained in the monodisperse range (PDI < 0.3) with no uniform trend observed, apart from a dramatic increase in PDI at N/P = 30 compared to N/P = 3, **P = 0.0012 (Fig. 3(a3)). This may be due to an increase in viscosity of the polymeric core stream at high N/P ratio^48^ and thus a wide residence time distribution (RTD) in the flow reactor. Zeta potentials were observed to increase with N/P ratio (Fig. 3(a4)), crossing neutral (0 mV) above N/P = 4, reaching up to 40 mV at N/P 30 because of the high concentration of polymer in the system.^44^ The trends observed in our work at lower N/P ratios (which are consistent with partial pDNA condensation) and charge of the formulation transitioning from anionic to cationic at higher N/P ratios are in accordance with many studies focusing on polymer-nucleic acid complexes. For instance, Erbacher *et al.*^49^ studied complexation of various PEI/DNA complexes and reasoned that at a particular N/P ratio, charge neutralisation occurs and reduces electrostatic stabilisation of the particle leading to larger sizes, observed in our case at ∼N/P = 3–4, where the zeta potential is also roughly neutral. It is noteworthy that the number of cells expressing GFP after treatment with nanogels synthesised at N/P = 3 was 2.5-fold higher than the commercial vector Lipofectamine 3000 (Fig. 4(a6)). An increase in complex size and charge neutralisation of the formulation up to a certain increase in N/P ratio is beneficial, as it has been proven to decrease the electrophoretic mobility of DNA, thus showing a strong complexation and increased DNA protection.^49,50^ However, at high N/P ratio (> 4), the nanogels acquired a very high cationic charge and some of the polymer remained as excess in the suspension confirmed from electron microscopy (Fig. S3(d and e)).

Due to the high cationicity and excess polymer, the formulations could become cytotoxic and lead to necrotic cell damages and membrane destabilisation.^51–53^ Accordingly, we found a reduction in the GFP expressed by nanogels synthesised at N/P ≥ 5 and none was observed for nanogels synthesised at N/P = 30 (Fig. S4, SI). While nanogels synthesised at N/P = 4 exhibited favorable physicochemical properties, nanogels synthesised at N/P = 3 were selected for further optimisation, as they had the lowest polymer-to-pDNA ratio, yielding a high GFP expression (better than the positive control). Additionally, they demonstrated excellent monodispersity (PDI = 0.14 ± 0.045), particle size below 200 nm (171 ± 27 nm) and a high pDNA encapsulation (93 ± 2%).

#### 3.1.2 Effect of cross-linker (TPP) concentration

The formation of spherical and compact nanogels is promoted by the anionic cross-linker TPP, which facilitates controlled gelation through ionic interactions with chitosan.^54^ This interaction neutralises the protonated amino groups on chitosan and leads to the formation of stable cross-linked networks.^55^ To assess the effect of TPP concentration on nanogel formation, we varied its concentration from 0.0047% to 0.095% W/V (% W/V = g/100 mL), while maintaining a fixed N/P ratio of 3 (in the reactor) by adjusting the initial polymer concentration. Final pDNA concentration was 25 µg mL^−1^ under reactor operating conditions of FRR = 0.1 and RT = 31 s. TPP concentrations are interchangeably referred to as −0.5× (0.0047%), 1× (0.0095%), 2× (0.019%), 5× (0.047%) and 10× (0.095%) in this study.

As the concentration of the cross-linker increased from TPP 0.5× to 10×, the corresponding phosphate molar ratio *P*_TPP_/*P*_pDNA_ increased from 5.1 to 103. This indicated a higher availability of TPP relative to the constant amount of pDNA, enabling the nanogels to encapsulate more genetic material within their polymeric structure. As a result, the observed near-complete DNA encapsulation (≥ 99%, Fig. 3(b2)) was likely due to increased ionic cross-linking between TPP, chitosan, and PEI,^50^ while the increasingly negative zeta potential (***P < 0.001, Fig. 3(b4)) could be attributed to excess TPP. At a low *P*_TPP_/*P*_pDNA_ = 5.1 (TPP 0.5×), the available TPP is likely insufficient for effective cross-linking and hence compaction. Correspondingly, at this low TPP concentration (0.0047% W/V), nanogels exhibited a size of 292 ± 93 nm as measured by DLS (Fig. 3(b1)), and a larger average particle size of 413 ± 101 nm based on TEM image analysis (*n* = 50, measured *via* ImageJ, Fig. S5(a), SI), indicating insufficient compaction. As the TPP concentration increased from 1–10× (*P*_TPP_/*P*_pDNA_ = 10.3 to ∼103), the average nanogel size initially decreased, reaching below 200 nm at TPP 1× (0.0095% W/V), then increased again at higher concentrations (Fig. 3(b1)). Fan *et al.* observed similar effects, attributing the size increase to additional chitosan attachment to excess TPP for the formation of a single nanoparticle.^55^ No statistically significant size or PDI differences were observed between 0.5× and higher concentrations, but GFP expression rose sharply at 1× (Fig. 4(b1, b2 and b6)), indicating a notable increase in transfection efficiency (**P < 0.0001). Huang *et al.* demonstrated that PEI–TPP complexes protect DNA by reducing its electrophoretic mobility, while exhibiting lower cytotoxicity and improved transfection efficiency compared to bPEI alone, due to charge neutralisation.^50^ However, at near-neutral zeta potential (−4.1 ± 2.5 mV) for TPP 0.5×, a low GFP expression was observed likely due to larger particle sizes (292 ± 93 nm). An increasingly negative zeta potential of −12.5 ± 0.3 mV and particle size of 171 ± 26 nm, achieved with TPP 1×, resulted in significantly higher transfection efficiency (Fig. 4(b6)).

Interestingly, despite DLS data indicating monodisperse particle sizes at TPP 2×, TEM images revealed a mix of small nanogels and large hexagonally shaped structures, likely due to rapid sedimentation not captured by DLS (Fig. S5(b, c) and S3(f)). Similar aggregates were also observed at TPP 5×, which may correspond to excess, uncross-linked TPP remaining in the dispersion. At TPP 10×, particle sizes increased significantly (**P < 0.01, Fig. 3(b1)), which could be attributed to higher TPP concentrations affecting chitosan membranes by increasing porosity, degree of swelling and cross-linking.^56^ These structural alterations and the presence of excess TPP observed at *P*_TPP_/*P*_pDNA_ ≥ 20.5, likely contribute to the decreased number of GFP-expressing cells at TPP 2× (0.019% W/V) and above (Fig. 4(b3–b6)), despite similar encapsulation efficiencies. These results indicate that TPP enhances cross-linking and nanogel compaction effectively up to a moderate concentration (TPP 1× here − *P*_TPP_/*P*_pDNA_ = 10.3). However, beyond this point, higher concentrations adversely affect formulation quality and significantly reduce transfection efficiency, as reflected in Fig. 4(b1–b6). Therefore, determining and maintaining an optimal TPP concentration is essential for designing an effective nanogel-based delivery system.

Based on these findings, TPP at 0.0095% W/V (1×) corresponding to a phosphate molar ratio of *P*_TPP_/*P*_pDNA_ ∼10.3 was selected as the optimal concentration. This formulation achieved target properties such as high DNA encapsulation, small nanogel size, and a high transfection efficiency.

#### 3.1.3 Effect of pDNA concentration

Higher pDNA loading in nanogels is beneficial, as it increases the amount of genetic material available per unit volume, specifically where high dosages are required. However, maintaining nanogel physicochemical properties and efficient gene delivery at elevated loadings is essential. As shown in Fig. 3(c1–c3), nanogels with desirable properties (size < 200 nm, PDI < 0.3, EE > 90%) were successfully synthesised at pDNA loadings ranging from 12.5–75 µg mL^−1^. Nnanogel formation in the presence of pDNA leads to condensation and collapse of the pDNA, resulting in smaller particles compared to those formed without pDNA (Fig. 3(c1)). At low pDNA loadings (12.5 µg mL^−1^), nanogels had relatively smaller sizes (125 ± 15 nm) and lower encapsulation (90.1 ± 3.3%) compared to higher pDNA loadings. TEM images in Fig. S5(e–h), show a lighter contrast for nanogels at 12.5 µg mL^−1^ and darker particles at higher pDNA loadings. This suggests that pDNA in lower amounts may have limited interactions with the polymer leading to lower cross-linking density of the network (thus particles of lighter shade were seen in TEM) and due to less reservoir space for entrapment also led to free (unencapsulated) pDNA in the system. At higher pDNA concentrations, increased electrostatic interactions promote greater polymer condensation, resulting in a denser nanogel network. This is evidenced by the reduction in nanogel anionic charge with increasing pDNA loading (Fig. 3(c4)), indicating greater utilisation of cationic polymer to encapsulate the available pDNA. Encapsulation efficiency exceeded 99% at loadings above 50 µg mL^−1^, further confirming this effect. However, beyond this threshold, the formation of high-density polymer networks led to increased particle size and aggregation (Fig. S5(g and h)). At 100 µg mL^−1^, nanogels exhibited elevated size and polydispersity (PDI > 0.3), likely due to uneven expansion of the polymeric cross-links as the network attempts to accommodate excess cargo. As a result, transfection ability was maintained up to 50 µg mL^−1^ but declined significantly at 75–100 µg mL^−1^ (Fig. 4(c1–c6)), possibly due to higher intra-nanogel interactions owing to higher amount of pDNA and stronger polymer network. Additionally, strong pDNA entrapment may influence drug release efficiency,^57^ suggesting that pDNA concentration can modulate release kinetics. At high loadings, complex interactions leading to uneven distribution and tightly packed structures, may hinder the release of genetic material from the hydrogel network, ultimately limiting nanogel performance as a gene delivery vector. Based on these findings, 12.5–50 µg mL^−1^ was identified as the optimal pDNA loading range that maintains effective *in vitro* gene delivery for the nanogel system studied.

### 3.2 Influence of reactor operation parameters

After determining the optimal formulation conditions for nanogels (N/P = 3, TPP = 0.0095% W/V, pDNA = 12.5–50 µg mL^−1^), we focused on optimising the reactor operating conditions, which play a critical role in maintaining nanogel properties in continuous flow and ensuring a successful scale-up. A pDNA concentration of 25 µg mL^−1^ was selected for further studies to reduce reagent consumption, given the high cost and laborious extraction and purification process of pDNA. Since the particle properties at this concentration were comparable to those at both the lower (12.5 µg mL^−1^) and higher (50 µg mL^−1^) bounds, 25 µg mL^−1^ represented a practical compromise.

#### 3.2.1 Effect of residence time (RT)

To investigate if there was sufficient time for reaction completion, the residence time in the coaxial flow reactor (CFR V3) was varied from 3 s to 31 s, where the core stream consisting of polymer mixed at a flowrate ratio of 0.1 with the sheath stream (final conc. of TPP = 0.0095% W/V, and pDNA = 25 µg mL^−1^, N/P = 3). As a result, the average Reynolds number, Re_avg_ (and the average velocity, *u*_avg_) in the co-axial reactor varied between ∼4–41 (0.0033–0.033 m s^−1^). For more information on the Re and fluid velocity, refer to Section S3 in the SI.

**Fig. 5:**
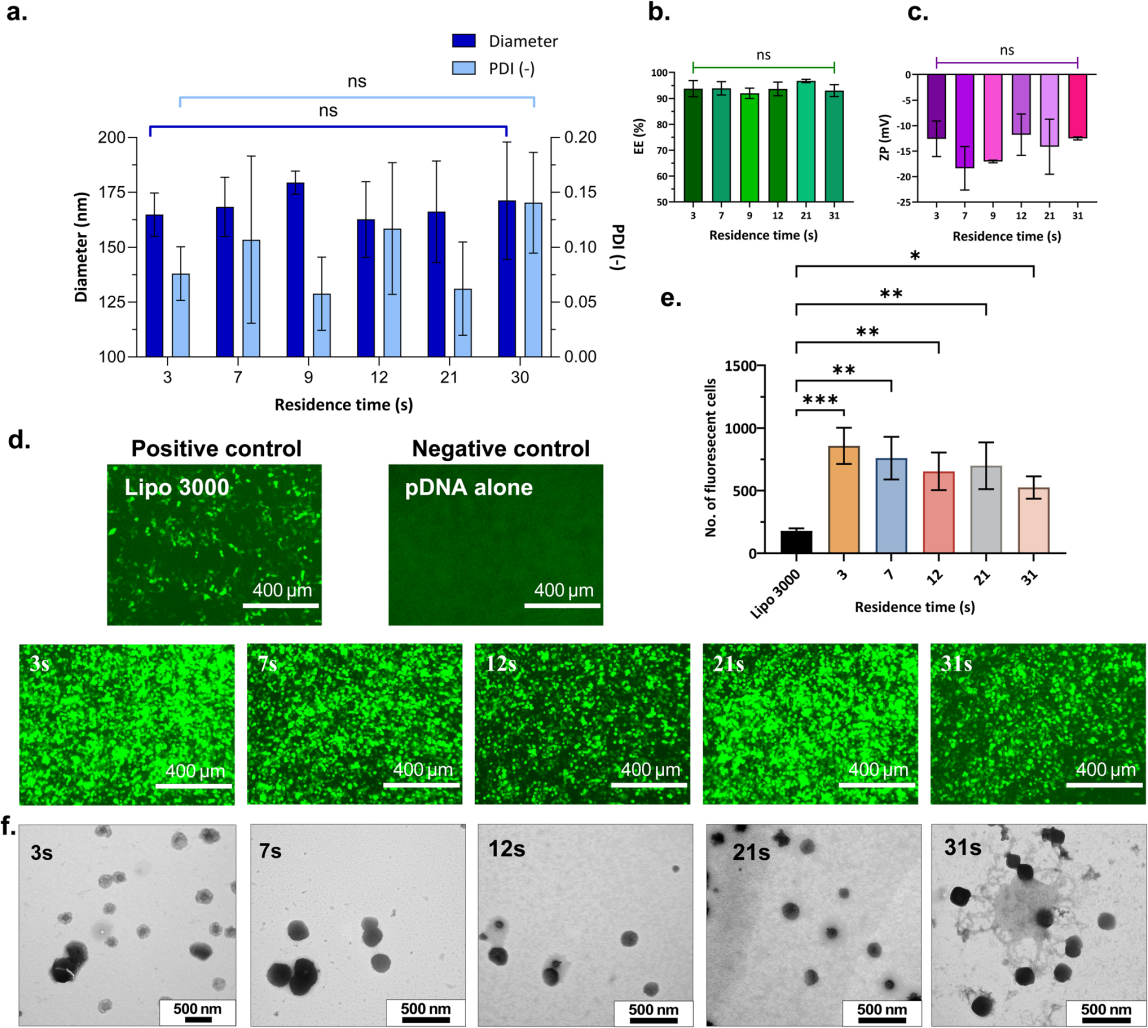
Physicochemical properties and transfection efficiency of nanogels produced with varying residence time (RT). (a) Nanogel size, (diameter) and polydispersity index, (PDI) *vs.* RT; (b) encapsulation efficiency, (EE) *vs.* RT; (c) zeta potential, (ZP) *vs.* RT. A one-way ANOVA study and Tukey's multiple comparisons test were performed to assess the effect of residence time on each of the dependent variables – diameter, EE, PDI and ZP, separately. Plots illustrate mean ± standard deviation and the results from Tukey's HSD, comparing the means of each RT with one another for each dependent variable; (d) fluorescence microscopy images of GFP expression in HEK293T cells at 72 h post-transfection shown for positive control (Lipofectamine 3000), negative control (pDNA without a vector) and nanogels synthesised with residence time of 3 s, 7 s, 12 s, 21 s and 31 s; (e) quantitative GFP assessment – number of fluorescent cells detected for positive control and nanogels produced at varying RT. A one-way ANOVA study and Dunnett's *post hoc* test was performed to compare GFP expression of positive control with nanogels prepared at varying RT; (f) TEM images of nanogels synthesised with varying RT. Nanogels were produced at N/P = 3, TPP = 0.0095% W/V, pDNA = 25 µg mL^−1^ and FRR = 0.1, in CFR V3. *P < 0.05, **P < 0.01, ***P < 0.001, ****P < 0.0001, ns = not significant (*P* > 0.05).

The nanogels formed by ionic gelation exhibited an average size below 200 nm and were monodisperse, with PDI < 0.2 across all residence times investigated (Fig. 5(a)). All formulations demonstrated high encapsulation efficiency (> 92%), Fig. 5(b). The observed anionic zeta potential (Fig. 5(c)) is likely due to the highly negatively charged pDNA^58^ and TPP, which neutralise the positive charge of the polymer during nanogel complexation. At a residence time of 3 s, nanogels with an average size of 164 ± 10 nm were obtained, which slightly increased to 171 ± 27 nm at 31 s. To verify reaction completion, the suspension collected after 10 s (for RT = 3 s) was stirred in batch at 500 rpm for an additional 5 min. No significant change in diameter was observed, suggesting that the cross-linking was complete within the microfluidic flow reactor. The rapid reaction completion (within 3 s) can be attributed to intensified mixing in the coaxial flow reactor (core = 0.14 mm, sheath = 1.12 mm). GFP expression and TEM images of nanogels synthesised at varying residence times are presented in Fig. 5(d and f). As shown, neither the transfection ability nor nanogel morphology significantly differed between nanogels produced at the lowest residence time of 3 s and at the highest residence time of 31 s. Moreover, the transfection efficiency of all nanogels exceeded that of Lipofectamine 3000 (Fig. 5(e)), irrespective of the residence time. These findings confirm that nanogel formation is effectively completed within 3 s under the tested conditions (N/P = 3, FRR = 0.1) in the microfluidic coaxial flow reactor.

To further investigate the effect of residence time, additional experiments were conducted at a higher N/P ratio of 12, while maintaining all other parameters constant. At this ratio, both nanogel size and encapsulation efficiency increased with residence time, reaching only 70% EE at the highest residence time of 31 s, whereas PDI and zeta potential did not show a significant dependency on the residence time (Fig. S6, SI). These observations suggest that a higher initial polymer concentration necessitates longer complexation times for reaction to complete. One contributing factor could be the increased viscosity of the polymer stream resulting in slower diffusion of the polymer and consequently affecting the mixing process. An increase in viscosity of solutions also delays the diffusion of DNA.^59^ Additionally, viscosity influences the diameter of the stream in a coaxial reactor,^60^ where the less viscous stream becomes thinner and the more viscous stream in the core expands towards the reactor wall. This may result in larger diffusion paths requiring higher mixing times in the reactor and a consequent reduction in the mixing quality under these conditions. Therefore, accurately estimating the mixing time in a coaxial flow reactor requires consideration of concentration (and viscosity) of both the reagent streams (hence, diffusion coefficients) in addition to the hydrodynamics in the reactor. Given that our earlier parametric study demonstrated effective GFP expression at a lower N/P ratio of 3 (**Section 3.1.1**), and that rapid complexation was achieved within 3 s under these conditions, this formulation was selected for further system optimisation.

#### 3.2.2 Effect of reagent feeds configuration

Nanogels produced in the study above possessed all the desired physicochemical characteristics, except the zeta potential which was still found to be anionic. Nanogels with cationic charge could be obtained either at high N/P ratios during synthesis or by coating the nanogel surface with an additional layer of cationic polymer post-synthesis. However, as discussed in **Section 3.1.1**, using higher polymer concentration reduces transfection efficiency and could result in cell death. Coating nanogels to modify surface charge is also undesirable as it adds an additional downstream processing step, and may result in the coating being stripped off when nanoparticles are subjected to shear stress from fluid forces in the blood stream.^61^ Therefore, we investigated the effect of swapping the reagents introduced in the core and sheath streams in the coaxial flow reactor to explore whether it could alter the nanogel characteristics.

**Fig. 6:**
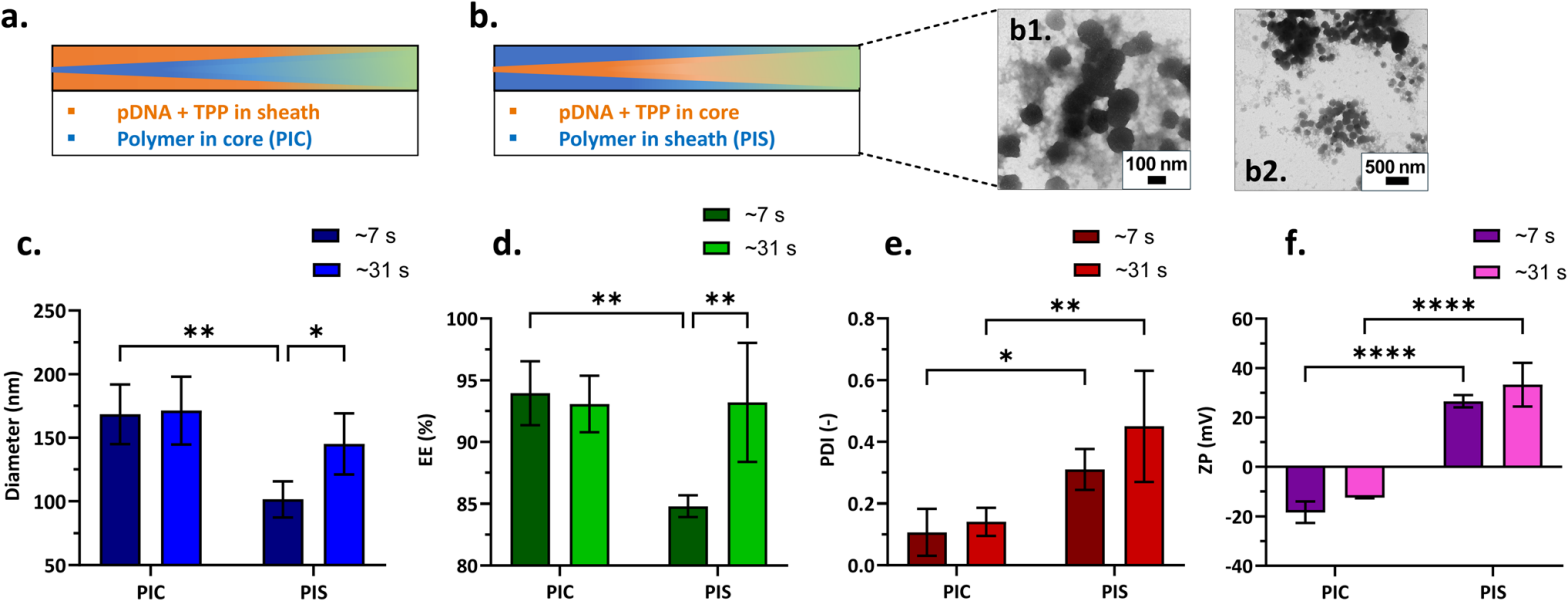
Graphical representation of reagent feeds configuration in coaxial flow reactor where polymer is represented in blue and pDNA + TPP in orange color: (a) polymer in core (PIC, original streams) configuration; (b) polymer in sheath (PIS, inverted streams) configuration and (b1 and b2) corresponding TEM images of nanogels synthesised with PIS configuration, FRR = 0.1. Variation in physicochemical properties of nanogels produced at residence time (RT) ∼7 and ∼31 s with different reagent feeds configurations: (c) nanogel size, (diameter); (d) encapsulation efficiency, (EE); (e) polydispersity index, (PDI); (f) zeta potential, (ZP). A two-way ANOVA study and uncorrected Fisher's LSD test was performed to assess the effect of feeds configuration and RT on each of the dependent variables – diameter, EE, PDI, and ZP, separately. *P < 0.05, **P < 0.01, ***P < 0.001, ****P < 0.0001. Nanogels were produced at N/P = 3, TPP = 0.0095% W/V, pDNA = 25 µg mL^−1^ and FRR = 0.1, in CFR V3.

In the new arrangement, the feeding location of cationic polymer was switched from the core to the sheath stream. This new arrangement (also referred to as ‘inverted streams’) consisted of CMC-bPEI polymer in the sheath stream, while pDNA and TPP were introduced in the core stream at a flowrate ratio (FRR = 0.1). Experiments using this configuration were conducted at two residence times (RT ∼7 and 31 s), with other optimised parameters held constant (pDNA 25 µg mL^−1^, TPP 0.0095% W/V, N/P = 3). Fig. 6 illustrates the feeds configurations, where the original arrangement – polymer introduced in core (PIC) is shown in Fig. 6(a) and the new configuration featuring inverted streams – polymer in sheath (PIS) is shown in Fig. 6(b). For a RT of ∼7 s, the implementation of PIS resulted in a reduction in the diameter and encapsulation efficiency and led to the formation of a highly polydisperse sample in the new configuration (Fig. 6(c–e)). As seen from Fig. 6(b1 and b2), the microscopy images displayed presence of aggregation along with small spherical particles in the suspension, thus indicating polydispersity. Moreover, it was interesting to note that, with an inversion of streams, the nanogel zeta potential changed from anionic to cationic without any change in stoichiometry (Fig. 6(f)). This tuneability of the overall charge in a coaxial reactor could be advantageous as it eliminates the need for an additional coating to render the nanogels cationic for systemic administration.

**Fig. 7:**
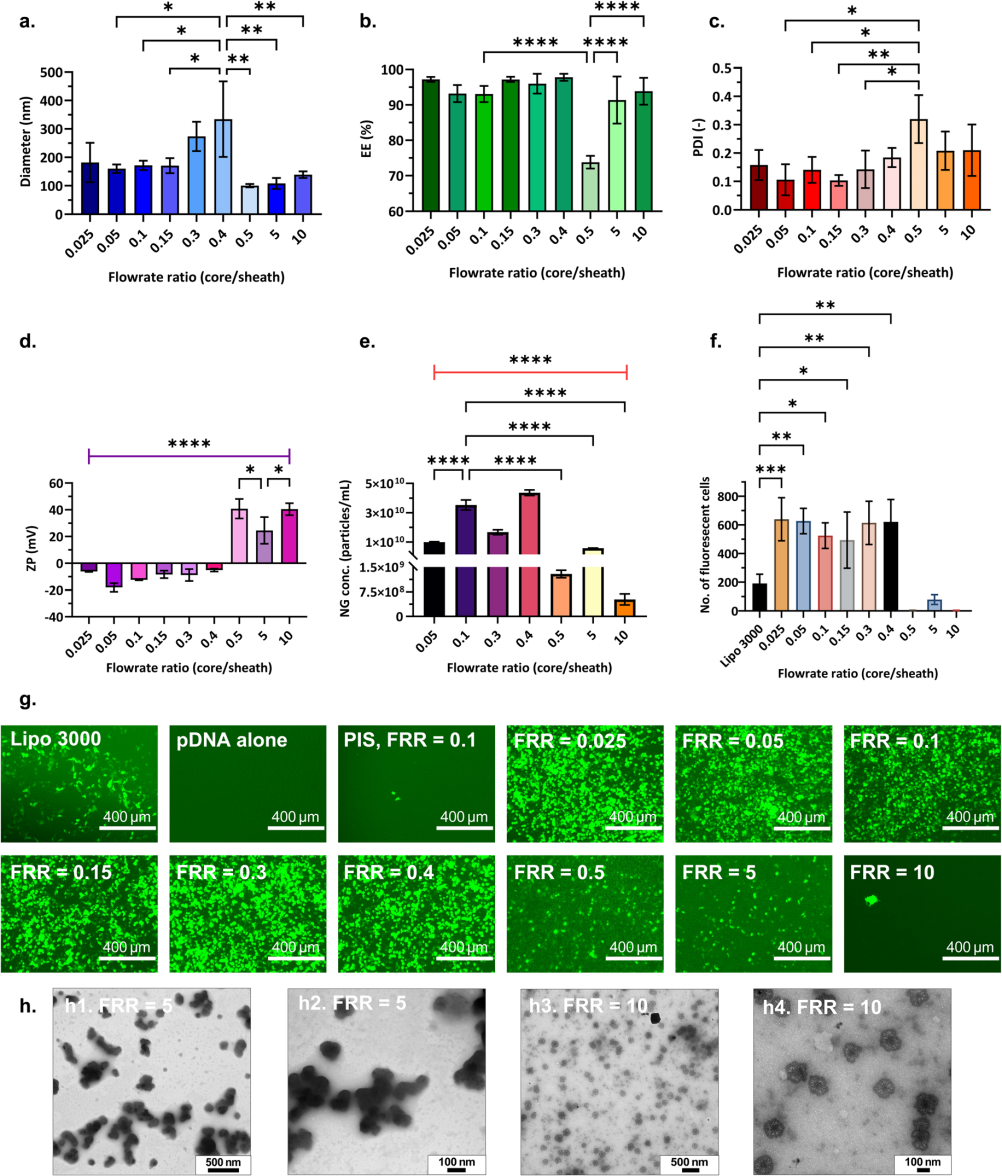
Physicochemical properties and transfection efficiency of nanogels produced with varying flowrate ratio (FRR). (a) Nanogel size (diameter) *vs.* FRR; (b) encapsulation efficiency, (EE) *vs.* FRR; (c) polydispersity index, (PDI) *vs.* FRR; (d) zeta potential, (ZP) *vs.* FRR; (e) particle concentration *vs.* FRR. A one-way ANOVA study and Tukey's multiple comparisons test were performed to assess the effect of FRR on each of the dependent variables – diameter, EE, PDI, ZP and particle concentration, separately. Plots illustrate mean ± standard deviation and the results from Tukey's HSD, comparing the means of each FRR with one another for each dependent variable; (f) quantitative GFP assessment – number of fluorescent cells detected for positive control and nanogels prepared at different FRR. A one-way ANOVA study and Dunnett's *post hoc* test was performed to compare GFP expression of positive control (Lipofectamine 3000) with nanogel formulations prepared at different FRR. *P < 0.05, **P < 0.01, ***P < 0.001, ****P < 0.0001; (g) fluorescence microscopy images of GFP expression in HEK 293T cells 72 h post-transfection for positive control (Lipofectamine 3000), negative control (pDNA without a vector) and nanogels synthesised at different FRR and feeds configurations; PIS refers to polymer in sheath, inverted streams. All nanogels were produced in CFR V3 at N/P = 3, RT = 31 s, pDNA = 25 µg mL^−1^, TPP = 0.0095% W/V and feeds configuration PIC, polymer in core, unless specified otherwise; (h) TEM images of nanogels synthesised at PIC, FRR = 5 and FRR = 10.

Even with unchanged stoichiometry, nanogel properties differed when the streams were interchanged. Apparently, the location of the diffusing stream in a coaxial reactor plays a vital role in the formation of nanogels. pDNA diffuses slowly, with a diffusion coefficient on the order of 10^−12^ m^2^ s^−1^ ^59,62^ (approximated for similar concentrations used in this study *i.e.* initial solutions: polymer < 2% W/V, pDNA < 110 µg mL^−1^). This is two orders of magnitude lower than the polymer itself (10^−10^ m^2^ s^−1^),^63–65^ as approximated for the bPEI MW 20 kDa (closest to that used in this study MW 25 kDa). In a fully developed laminar flow profile, due to friction at the walls, the fluid elements in the center move faster than those near the wall. When the slowest diffusing molecule (pDNA) is placed in the core stream and inevitably at the centerline of laminar flow, it has a lesser chance to diffuse and mix in the reactor. This could lead to incomplete mixing within the reactor; mixing would continue under uncontrolled conditions after the product is collected, thus leading to nanogel aggregation and a rise in PDI. Irrespective of the residence time used, the feeds configuration has a pronounced effect on nanogel polydispersity, PDI being higher for PIS feeds configuration as can be seen from Fig. 6(e). Furthermore, the two-way ANOVA study and uncorrected Fisher's LSD revealed that in the PIS feeds configuration, with an increase in RT from ∼7 to ∼31 s, a significant rise in the diameter (*P = 0.0457) and encapsulation efficiency (**P = 0.0090) of the nanogels was observed. Therefore, a residence time of 31 s was probably not sufficient for complete diffusion of pDNA in the new arrangement. Consequently, it is recommended that molecules with faster diffusion rates should be positioned at the center of the coaxial reactor, while the slower diffusing molecules be allocated to the sheath stream, where the velocities are lower, to prevent incomplete mixing within the coaxial flow reactor. To investigate the effect of reagent feeds configuration further, we performed nanoparticle tracking analysis (NTA) of nanogels formed with both feeds configurations – PIC and PIS. It was found that the average concentration of particles formed when streams were inverted (PIS) was a mere ∼1.08 × 10^8^ particles per mL, significantly lower than concentrations obtained when polymer flowed in the core (PIC) ∼3.53 × 10^10^ particles per mL. One might argue that the encapsulation efficiency for nanogels produced at RT ∼31 s in both the configurations was similar, but the particle concentration was very low in the new arrangement. It is possible that uncontrolled mixing occurring at the outlet of the reactor led the free pDNA to complex with unreacted components from the reactor and form complexes, but particles were not necessarily formed. Hence, the measured unencapsulated pDNA was comparable, but these irregular complexes were not detected in NTA, thus leading to low nanogel concentration in PIS feeds configuration. However, a more comprehensive investigation is required to further clarify and better understand these effects. The cationic charge measured in zeta potential measurements was most likely due to the excess unreacted polymer in the suspension, which could be attributed to incomplete mixing in the reactor. Furthermore, low to no GFP expression was observed in transfection experiments for formulation produced with PIS feeds configuration at FRR = 0.1 (Fig. 7(g)), which may be attributed to the high local concentration of the uncross-linked free polymer as well as low particle concentration in the nanogel suspensions produced when streams were inverted. Hence, feed locations were switched back to their original arrangement – feeds configuration PIC, as demonstrated in Fig. 6(a) for further studies.

#### 3.2.3 Effect of flowrate ratio (FRR)

To determine whether quality of nanogels produced could be improved further, the effect of mixing was investigated in CFR V3. The flowrate ratio determines how fast the core stream flows relative to the sheath stream. In a coaxial reactor, mixing efficiency can be improved by increasing the flowrate of the inner (core) stream. Under these conditions, albeit a fully turbulent regime (considered beneficial for rapid nanoparticle synthesis) is not attained at low Re, mixing is still enhanced due to microvortex formation.^66,67^ It has been proposed that operation with FRR < 0.01 (and hence low core stream velocity) is undesirable for nanoparticle synthesis, as mixing is difficult to quantify.^66^ Therefore, the effect of flowrate ratio was investigated by increasing the ratio of volumetric flowrate of core stream to the sheath stream in CFR V3 from 0.025 to 10. Accordingly, as the core flowrate increased and sheath flowrate decreased for an increase in flowrate ratio, the velocity ratio (VR = core stream velocity : sheath stream velocity) also increased from 1.1 to 456.3. A residence time of 31 s in the reactor was maintained to allow sufficient reaction time for nanogel formation. Hence, Re_avg_ and *u*_avg_ were fixed at ∼4.08 and ∼0.0033 m s^−1^ respectively for all flowrate ratios studied. Additional data on calculations of Reynolds numbers and fluid velocities of individual streams are available in Section S3, in the SI. All other parameters were constant at pDNA loading = 25 µg mL^−1^, N/P = 3, TPP concentration 0.095% W/V and RT = 31 s. The initial concentration of polymer in the syringes was adjusted according to the flowrate ratio to obtain a polymer concentration of N/P = 3 in the reactor (after the two streams mixed).

Fig. 7(a) demonstrates an increase in nanogel diameter with an increase in the flowrate ratio up to 0.4, after which (at FRR ≥ 0.5) nanogel diameters dropped below 150 nm, with a slight increase in polydispersity (Fig. 7(c)). Encapsulation efficiency remained above 90% across most conditions Fig. 7(b), additionally, it was interesting to note the zeta potential transformation from anionic to cationic at higher flowrate ratios (Fig. 7(d)). Further investigation carried out using NTA measurements showed that at higher flowrate ratios (FRR ≥ 0.5), nanogel formation was significantly lower than those formed at lower flowrate ratios (< 0.5) (Fig. 7(e)). The cationicity observed in these formulations could be contributed by free unreacted polymer as discussed in **Section 3.2.2**. Even though the total polymer content delivered to the reactor remained the same, there would be slight variation in the overall viscosity of the solutions because the initial polymer concentration in the syringe was varied to accommodate each flowrate ratio and maintain N/P = 3 in the reactor. Due to this, it is possible that varying concentration gradients were created in the reactor that would affect the overall rate of polymer diffusion and hence the mechanism of formation of nanogels.

Lim *et al.*^66^ demonstrated in a coaxial mixer, with a core (23G needle, typical ID = 0.33 mm, OD = 0.65 mm) ∼2.35× larger than used in this work, that the mixing time is affected as the velocity ratio increases at low Re_avg_ (< 400–500). Operating the mixer in a turbulent regime, at high Re_avg_ (> 500) and low mixing times, stable, monodisperse iron oxide nanoparticles, PLGA-PEG nanoparticles and siRNA-PEI polyplex nanoparticles, among others were synthesised. When operating the reactor with a high core stream flowrate, Re_core_ increases leading to an increase in mixing efficiency. However, for our case with an increase in the velocity ratio, although Re_core_ increased up to 456, the Re_avg_ was still very low (∼4.1), hence the reactor was still operating in the laminar regime. The flow may be stratified, which might have led to a slow and incomplete mixing of the fluid. Beyond a critical point of flowrate ratio, flow recirculation develops. As shown by Baber *et al.*^68^ where the core ID was 0.798 mm, as Re_core_ increased, the jetting of core stream transitioned to a complete spread across the entire cross section of the channel through a recirculation pattern. It might be possible that for CFR V3, this critical point is between FRR = 0.4 and 0.5, thus leading to an abrupt change in behaviour, apparent from nanogel properties. Up to FRR = 0.4 there might be no recirculation, the core thickness keeps increasing, so mixing time increases, leading to larger nanogels. Beyond FRR = 0.4, even if there is recirculation, the diffusion of DNA through the lamella created is slower. This might have led to a rise in the PDI at FRR = 0.5 (Fig. 7(c)) because uncontrolled mixing would have occurred in the droplets at the outlet of the reactor. The slow diffusion would further explain the decrease in the concentration of nanogels produced at high flowrate ratios (Fig. 7(e)).

Fig. 7(f and g) demonstrates GFP expressed by nanogels produced at varying flowrate ratios. Samples of nanogels produced at lower flowrate ratios (with high particle concentration) displayed significant transfection ability, as exhibited by their crowded GFP expressions. It was interesting to note that for the lowest FRR = 0.025, although we operated very close to the velocity ratio of 1 (VR = 1.1), the nanogels synthesised even at low Re_avg_ were in the desired range and showed good transfection, thus demonstrating that operation in laminar regime is suitable for pDNA nanogels. It was evident that anionic nanogels produced at FRR < 0.5 performed substantially better than cationic nanogels produced at FRR ≥ 0.5. While of the same order of magnitude, the particle concentration for nanogels produced at FRR = 5 was about 1.6× lower (∼5.8 × 10^9^ particles per mL) when compared to nanogels produced at FRR = 0.05 (∼9.23 × 10^9^ particles per mL). Hence, although cationic nanogels below 200 nm were produced at FRR = 5, the presence of aggregation (Fig. 7(h1 and h2)) and low particle concentration could be contributing factors to low GFP expressed in HEK293T cells. The particle concentration further dropped to 5.2 × 10^8^ particles per mL at FRR = 10, and simultaneously highly cationic nanogels (40.42 ± 4.5 mV) were obtained. Fig. 7(h3 and h4) displays TEM of nanogels produced at FRR = 10. A high positive charge of bPEI and its polyplexes has been shown to be toxic to cells and reduces cell viability.^20,69–71^ The combined effects of high cationicity and low particle concentration likely led to the poor transfection observed for these nanogels, as illustrated in Fig. 7(f and g). Therefore, successful nanogel production depends not only on optimising their physicochemical properties but also on achieving high particle concentrations (of the order 10^9^–10^10^ particles per mL for this system).

The enhanced transfection of anionic CMC-bPEI-pDNA nanogels may be seemingly surprising, as positively charged nanoparticles are traditionally considered favourable for gene delivery due to electrostatic attraction to the negatively charged cell membrane. However, surface charge alone does not reliably predict transfection efficiency.^72,73^ Transfection outcomes are governed by a complex interplay of physicochemical and biological factors, including particle size, surface charge, morphology, particle concentration and cellular environment.^72,74^ In serum containing cell culture medium, the complex mixture of plasma proteins and other biomolecules in fetal bovine serum can significantly influence the stress response of cultured cells^75^ as well as modulate their gene expression.^76,77^ In this context, it is likely that serum proteins from the medium adsorb onto nanogels, forming a protein corona.^78^ Such corona formation can mask the native negative charge of the nanogels, shift their effective zeta potential toward neutrality or slight cationicity,^79^ and may present adsorbed proteins that interact with cell-surface receptors, potentially facilitating endocytosis.^80,81^ Particle concentration also plays a crucial role: higher local particle densities at the cell interface increase the probability of membrane interactions and uptake events.^82^ In our study, anionic nanogels had higher particle concentrations than cationic formulations, consistent with previous findings in HEK293T-derived exosomes, where higher concentrations of 10^9^ particles per mL enhanced cellular responses compared to lower concentrations (10^8^ particles per mL).^83^ Moreover, anionic nanogels also benefit from improved stability in physiological media, lower cytotoxicity^84,85^ and enhanced adhesion and permeation through the cell membrane.^73,84,86^ While the factors stated above enhance cellular uptake, the bPEI-mediated proton sponge effect,^69,87^ which facilitates endosomal escape and the subsequent release of encapsulated pDNA into the cytoplasm, is the critical intracellular mechanism ensuring high gene expression. Collectively, the optimal particle size and structure, serum-mediated protein corona formation, high particle concentration, reduced toxicity and efficient endosomal escape likely lead to the superior transfection performance of anionic nanogels in HEK293T cells.

Overall, we found that nanogels with target properties were successfully synthesised at high concentration under optimised operating conditions using the PIC feeds configuration, with slower-diffusing components placed in the sheath stream. Optimal mixing occurred at low flowrate ratio (FRR = 0.1), where the core stream diameter is minimised. These findings underscore the importance of optimising process parameters for effective scale-up.

### 3.3 Influence of reactor design and investigation of scalability

The identification of optimal formulation and reactor operating conditions (pDNA = 25 µg mL^−1^, TPP = 0.00954% W/V, *P*_TPP_/*P*_pDNA_ molar ratio ∼10, N/P = 3, PIC feeds configuration, FRR = 0.1, lowest residence time for nanogel formation ∼3 s) paved the way for developing a scalable pathway for nanogel synthesis that preserved physicochemical properties and *in vitro* gene delivery efficiency.

#### 3.3.1 Effect of capillary diameter and length

To increase the overall production capacity of a system, one way to scale-up is by increasing the length (*L*) and/or diameter (*D*) of the reactor to increase volume.^88^ To determine the scaling approach, the influence of sheath/core capillaries diameter and reactor length was investigated. The optimised conditions were employed in CFR V1–V2 (increase in reactor diameter) and CFR V4–V5 (increase in reactor length). The residence time was initially fixed at 7 s.

**Fig. 8:**
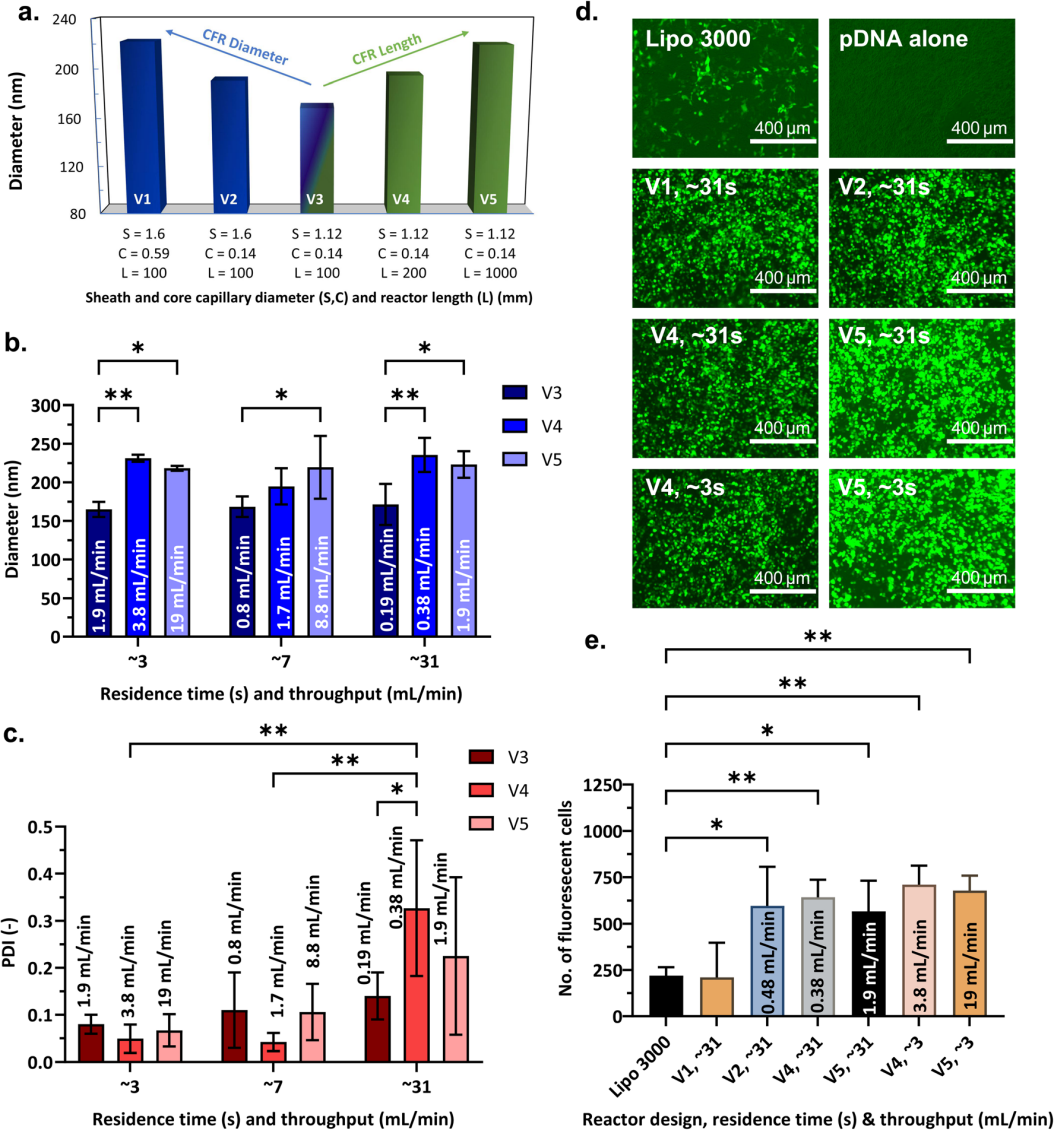
Scalability assessment of the coaxial flow reactors (CFRs). (a) Effect of reactor length and (core and sheath capillary) diameter on nanogel diameter, nanogels produced at residence time, RT = 7 s; (b) nanogel size, (diameter) *vs.* RT; (c) polydispersity index, (PDI) *vs.* RT; corresponding reactor throughputs are indicated within/above the bars; a two-way ANOVA study and Tukey's multiple comparisons test were performed to assess the effect of RT and throughput on each of the dependent variables – diameter and PDI, separately; (d) fluorescence microscopy images of GFP expression in HEK 293T cells 72 h post-transfection for positive control (Lipofectamine 3000), negative control (pDNA without a vector) and nanogels synthesised in different CFRs, RT specified in the figure for each respective CFR; (e) quantitative GFP assessment – number of fluorescent cells detected for positive control and nanogels produced with various CFR designs, RT (s) and throughputs (mL min^−1^). A one-way ANOVA study and Dunnett's *post hoc* test was performed to compare GFP expression of positive control with formulations prepared with different CFR designs and throughputs. Plots illustrate mean ± standard deviation and the results from *post hoc* tests. *P < 0.05, **P < 0.01, ***P < 0.001, ****P < 0.0001. Nanogels were produced at N/P = 3, pDNA = 25 µg mL^−1^, TPP = 0.0095% W/V, FRR = 0.1, and CFR designs and RT as specified.

Fig. 8(a) demonstrates the change in nanogel diameter as the reactor length and diameter is increased. When using a core capillary of larger diameter (0.59 mm, CFR V1), nanogels of 222 ± 113 nm were produced as opposed to 190 ± 54 nm obtained in lower diameter core capillary of 0.14 mm, CFR V2. A similar trend was seen in nanogels produced at RT = 31 s (Fig. S8(a), SI). Decreasing core and sheath capillary diameters has shown nanocomplex size reduction in a 3D-printed flow focusing device.^89^ Similarly, a reduction in the nanoparticle size with smaller inner capillary diameter was observed by Baber *et al.* for the synthesis of silver nanoparticles, which was attributed to the decrease in the diffusion distance across the stream caused by reduced thickness of the inner stream.^68^ A broader RTD occurring due to a larger core diameter, results in a rise in the polydispersity.^90^ However, a one-way ANOVA study performed to assess the effect of reactor diameter on nanogel diameter and PDI, produced at RT = 31 s, revealed no statistical differences in the measured properties (Fig. S8(a and b), SI). Nevertheless, nanogels with the lowest mean diameter were produced in CFR V3, with average size of 168 ± 23 nm and PDI = 0.07 ± 0.03. Hence, the core capillary ID = 0.14 mm and the sheath capillary ID = 1.12 mm were deemed preferable to produce nanogels < 200 nm and investigate the effect of capillary length.

Upon doubling the reactor length (CFR V4, reactor volume ∼0.2 mL) we found a slight increase in nanogel size (195 ± 23 nm), which further increased to 219 ± 41 nm on increasing the reactor length by a factor of 10 (V5) (Fig. 8(a)). The increase in nanogel size with reactor length was statistically significant for all residence times, as seen from Fig. 8(b). Adding a straight tube to increase the reactor length would lead to the possibility of Taylor dispersion that may increase polydispersity. To mitigate this and reduce the reactor footprint, the PTFE tube in CFR V5 was coiled to induce secondary flow and form Dean flow vortices, which would improve the radial mixing in the channel and lead to a narrower RTD. With Dean numbers being above the threshold of 1.5 for secondary flow to fully develop^91^ (additional data can be found in Section S3, in the SI), and calculated to be 43.26, 19.92 and 4.38 for RT of 3, 7 and 31 s respectively, it is expected that a fully developed Dean flow was achieved in the additional coiled reactor length (loop of 0.8 m) in V5. The decrease in the PDI at higher flowrates (Fig. 8(c)) indicated a pronounced effect of this secondary flow.

Nanogels with the desired properties were produced irrespective of reactor diameter at all residence times (3, 7, 31 s) – whereas for reactor length extension, optimal nanogels were produced only at higher throughputs and lower residence times (3, 7 s). Specifically, nanogel sizes remained consistent and well below 240 nm, with PDI < 0.3 and EE > 90% for nanogels produced in CFR V1 to CFR V3 for the residence time of 31 s (Fig. S8(a and b), SI). Similar results were observed at higher throughputs (RT ∼3 s and ∼7 s) for CFR V4 and CFR V5 (Fig. 8(b, c) and S8(c, d) in the SI).

#### 3.3.2 Scale-up

For microfluidic systems to be viable for large-scale manufacturing, the production process must be scalable while retaining the advantages of lab-scale systems. Increasing the throughput from 0.19 to 1.9 mL min^−1^ (RT = 3–31 s) in a 0.1 mL reactor volume (CFR V3) did not significantly affect the physicochemical properties or transfection performance of the nanogels as discussed in **Section 3.2.1**. Based on this result, a scalability study was conducted using larger reactors: V4 (0.2 mL) and V5 (1 mL), to assess whether the increased scale would compromise nanogel transfection efficiency. Accordingly, the throughput was increased by factors of 2 and 10 in CFR V4 (0.38–3.8 mL min^−1^) and CFR V5 (1.9–19 mL min^−1^), respectively. This represented a 100-fold scale-up from the smallest reactor volume to the largest (CFR V3–V5, 0.19 to 19 mL min^−1^). Nanogel properties produced at these conditions are shown in Fig. 8(b and c). As the throughput increased (and RT decreased), although sizes remained statistically unchanged, monodispersity of the formulations improved significantly. With an increase in reactor length and corresponding reactor volume, Re_avg_ rose from 4.08 to 41.05 in CFR V3, 8.21 to 81.03 in CFR V4 and 41.04 to 405.16 in CFR V5, corresponding to RT ∼31 s and ∼3 s, respectively. This trend indicates a higher degree of mixing with an increase in overall throughput. At the highest RT ∼31 s, the PDI was highest which could be attributed to higher axial dispersion in the laminar flow reactor, since it was operating at a low Re_avg_ indicating low degree of mixing. However, as seen from Fig. 8(d and e), transfection studies revealed that nanogels produced in reactor versions V2, V4 and V5, despite being negatively charged and exceeding 200 nm in size, exhibited excellent transfection efficiency. Nanogels obtained by CFR V1 still showed comparable transfection to positive control. Encapsulation efficiency exceeded 90% in all cases, and particle concentrations were close to the order of 10^10^ particles per mL (Fig. S8(b–e), SI), with CFR V3 yielding the highest concentration. Thus, the smallest inner diameters of the core and sheath capillaries result to the best nanogel quality, as indicated by smaller particle sizes and higher particle concentrations. Despite slight increases in nanogel size with scale-up, the successful 100× throughput increase confirmed that continuous-flow nanogel production can be scaled effectively while preserving product quality. This was achieved through reactor length extension and careful capillary selection.

An important factor to consider during scale-up is the rheological properties of the feed streams, as they influence mixing within the coaxial flow reactor. At the initial concentrations used in this work (< 2% W/V polymer; < 110 µg mL^−1^ pDNA), both polymer and pDNA solutions behave as nearly Newtonian, with diffusion being the dominant mixing mechanism under laminar flow. However, at higher polymer loadings or during scale-up, shear-thinning and viscosity-dependent deviations from Newtonian flow may affect micromixing and alter velocity profiles within the core and sheath streams, potentially slowing diffusion across the interface.^92^ In such cases, rheological characterisation of feed streams and computational fluid dynamics (CFD) modelling would be useful to guide reactor design and operating conditions.

Although flow focusing devices have been utilised to produce gene delivery materials,^40,41,89^ their reported throughputs while maintaining desirable material characteristics remain substantially lower than the 1.14 L h^−1^ achieved in this study. For instance, lipid–polymer hybrids^40^ and siRNA nanocomplexes^89^ have been produced at throughputs of 0.66 L h^−1^ and 0.18 L h^−1^, respectively. Encapsulation efficiencies also tend to be lower; for example, hyaluronic acid nanogels have previously demonstrated encapsulation efficiency of only 71%,^41^ whereas the formulations developed in this work achieved encapsulation efficiencies exceeding 90%. To further increase the overall process throughput, a feasible strategy is internal numbering up of the coaxial flow reactor. This can be accomplished by increasing the number of channels (while the channel length and diameter remain the same^88^). Uniform distribution of reactants across these channels using a single set of pumps and a centralised control system would enable consistent flow and scalable nanogel production.

## 4 Conclusions

In this study, we synthesised polymeric nanogels (CMC-bPEI-pDNA NGs) in a microfluidic coaxial flow reactor using a conjugated polymer – carboxymethyl chitosan grafted onto branched polyethyleneimine (CMC-bPEI) – for plasmid DNA delivery. This system leverages the synergistic benefits of chitosan and polyethyleneimine while mitigating their individual limitations. While CMC-bPEI has predominantly been utilised in batch systems, this study is among the first to investigate its continuous-flow microfluidic optimisation and scale-up for non-viral pDNA delivery. Through a systematic parametric study, we optimised nanogel synthesis in flow, investigating the roles of residence time, flowrate ratio, N/P ratio, cross-linker concentration and pDNA loading. Additionally, reactor engineering parameters such as hydrodynamics, reagent feeds configurations and reactor dimensions were investigated for their impact on nanogel quality. To our knowledge, this is the first detailed study on continuous-flow optimisation of pDNA nanogels.

Our in-house fabricated coaxial flow reactor enabled nanogel production with remarkable efficiency at a fraction of the cost of conventional microfluidic systems (∼£50 *vs.* > £350). This process achieved particle sizes below 200 nm, with excellent uniformity (PDI < 0.2), and > 90% pDNA encapsulation in just 3 s. These target properties were accomplished at a low N/P ratio = 3 and a moderate TPP concentration of 0.0095% W/V with a phosphate molar ratio *P*_TPP_/*P*_pDNA_ = 10. Nanogels could be loaded with high pDNA content (up to 50 µg mL^−1^) without compromising delivery performance. Key process insights revealed that placing the slowest diffusing components (pDNA and cross-linker) in the sheath stream and operating at a low flowrate ratio (core/sheath) enhances mixing efficiency, resulting in nanogels with optimal properties. Formulation zeta potential changed from anionic to cationic when adjusting the flowrate ratio (FRR > 0.5) or switching stream positions, potentially eliminating the need for post-synthesis modifications. However, cationic nanogels showed reduced particle concentrations, increased aggregation, and excessive charge, making anionic formulations more favorable for *in vitro* performance. We found that both nanogel physicochemical properties and particle concentration influence the transfection efficiency, with anionic nanogels offering superior performance. Our nanogels exhibited excellent *in vitro* transfection efficiency in HEK293T cells, outperforming commercial Lipofectamine 3000, as shown by fluorescence microscopy. Few polymeric systems in literature have achieved such performance. Nonetheless, further validation using flow cytometry, stability studies and *in vivo* models is required to confirm clinical relevance.

We successfully demonstrated scalability through careful capillary selection, reactor length extension, and increasing the throughput by a factor of 100 (0.19–19 mL min^−1^). This scaling demonstrated that nanogel production at larger scale maintained similar quality: particle size, surface charge, monodispersity, as at smaller scales. Importantly, gene delivery remained successful even at high throughputs of 100×, highlighting the robustness, reproducibility and industrial potential of the process. While coaxial reactors are not novel, their application for process-intensified synthesis of polymeric nanogels remains largely unexplored. This study bridges nanogel formulation and reactor engineering, offering a cost-effective, scalable platform for high throughput nanogel production for non-viral gene delivery. Our findings enhance the understanding of polymeric nanogel manufacturing in laminar flow systems and support the design of efficient, scalable processes for non-viral gene therapy materials.

## Author contributions

Suneha Patil – conceptualisation, methodology, formal analysis, investigation, data curation, visualisation, writing: original draft, review and editing, project administration; Zoe Whiteley – methodology, supervision, validation, review; Esther Osarfo-Mensah – investigation; Arun Pankajakshan – software; Duncan Q.M Craig – conceptualisation, resources; Stefan Guldin – review; Pratik Gurnani – supervision, validation, writing: review and editing; Asterios Gavriilidis – supervision, funding acquisition, resources, writing: review and editing.

## Conflicts of interest

There are no conflicts of interest to declare.

## Abbreviations

ANOVA: Analysis of variance
bPEI: Branched polyethylenimine
CFR: Coaxial flow reactor
CMC: Carboxymethyl chitosan
DLS: Dynamic light scattering
EE: Encapsulation efficiency
FRR: Flowrate ratio (ratio of volumetric flowrate of the core to the sheath stream)
GFP: Green fluorescent protein
ID: Inner diameter
LSD: Least significant difference
mRNA: Messenger RNA
MW: Molecular weight
N/P ratio: Molar ratio of nitrogen in the polymer to phosphate in pDNA + TPP
NFW: Nuclease free water
NG: Nanogel
NTA: Nanoparticle tracking analysis
OD: Outer diameter
PDI: Polydispersity index
pDNA: Plasmid DNA
*P*_TPP_/*P*_pDNA_: Molar ratio of phosphates in TPP and pDNA
PIC: Polymer in core stream
PIS: Polymer in sheath stream
RT: Residence time
siRNA: Small interfering RNA
TEM: Transmission electron microscopy
TPP: Sodium tripolyphosphate
Tukey's HSD: Tukey's honestly significant difference test
VR: Velocity ratio (ratio of velocity of the core to the sheath stream)
ZP: Zeta potential

## Supplementary Material

NA-008-D5NA00558B-s001

## Data Availability

The code used for quantitative GFP assessment can be found at https://github.com/arun-pn/blob-detector and has been archived with https://doi.org/10.5281/zenodo.15592498. All raw data can be obtained from the corresponding author upon request. Supplementary information (SI): additional material on coaxial flow reactor assembly, polymer conjugation procedure, pDNA extraction, transfection protocols, quantitative assessment of GFP expression, nanoparticle tracking analysis, as well as further details on nanogel optimisation, reactor hydrodynamic parameters, transfection experiments, reactor scalability and nanogel stability. See DOI: https://doi.org/10.1039/d5na00558b.
