# Supplementary material for: Microfluidic engineering of pDNA nanogels in a coaxial flow reactor: process development, optimisation, scalability and in vitro performance
Source: Nanoscale Adv. 2025 Oct 22;8(1):240–59. doi: 10.1039/d5na00558b (PMC12666657; doi:10.1039/d5na00558b)
Supplement: NA-008-D5NA00558B-s001 [file NA-008-D5NA00558B-s001.pdf]

Supplementary Information (SI) for Nanoscale Advances.
This journal is © The Royal Society of Chemistry 2025

Supplementary Information

Microfluidic engineering of pDNA nanogels in a coaxial flow reactor: process development, optimisation, scalability and *in vitro* performance

Suneha Patil, Zoe Whiteley, Esther Osarfo-Mensah, Arun Pankajakshan, Duncan Q. M. Craig, Stefan Guldin, Pratik Gurnani and Asterios Gavriilidis

Contents

S1. Methods 2
  a. Coaxial flow reactor assembly 2
  b. Polymer (CMC-bPEI) conjugation procedure 2
  c. Plasmid DNA amplification and extraction 3
  d. Details of transfection experiments 3
  e. Quantitative assessment of GFP expression from fluorescence microscopy images 4
  f. Nanoparticle tracking analysis 5
S2. Optimisation of nanogel properties for gene delivery 6
S3. Hydrodynamic parameters in the coaxial flow reactor 8
S4. Effect of pDNA amount, reagent feeds configuration, flowrate ratio on transfection experiments 10
S5. Effect of reactor design and investigation of scalability 11
S6. Nanogel stability 13
References 14

S1. Methods

a. Coaxial flow reactor assembly

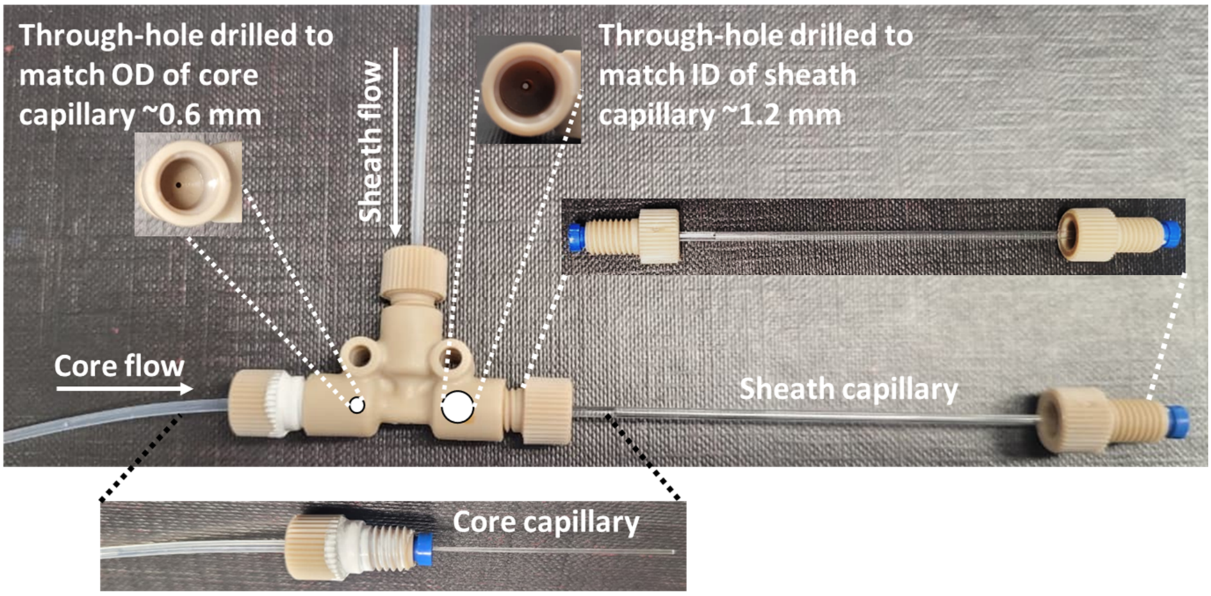

Figure S1. Coaxial flow reactor (CFR V3) assembly.

b. Polymer (CMC-bPEI) conjugation procedure

Carboxymethyl chitosan (CMC, Cruz Biochemicals) of medium molecular weight (210–300 kDa) was conjugated with branched polyethylenimine (bPEI, 25 kDa, Sigma Aldrich) using 1-ethyl-3-(3-dimethylaminopropyl) carbodiimide (EDC, Thermo Fisher Scientific) and N-hydroxy succinimide (NHS, Sigma Aldrich). The conjugation procedure for the polymer was adapted from Park et al[1] and Whiteley et al[2]. Solution 1 was prepared by dissolving 220 mg of CMC in 30 mL de-ionised (DI) water. Then, 30 g of bPEI was added to this solution and stirred for 30 min. The pH of the mixture was then adjusted to 5.5 (with 1 M HCL or 1 M NaOH). In another glass vial, solution 2 was prepared by dissolving 192 mg EDC and 115 mg NHS in 10 mL DI water. Solution 2 was then added dropwise to solution 1, under continuous stirring of 300 rpm. EDC and NHS aid in the covalent binding between carboxymethyl derivatives of chitosan and amine groups in bPEI[1,3]. After addition, the mixture was kept under stirring at room temperature for 24 h. The setup was covered with aluminium foil to avoid light interference as EDC is sensitive to light. The stirred solution was then dialysed (40 mL per 2 L DI water) for 2 days using a 7000 molecular weight cut-off (MWCO) snakeskin dialysis membrane (Thermo Fisher Scientific) with the DI water replaced after 24 h. The conjugated polymer (solution left in the dialysis bag) was then freeze dried, redispersed in nuclease free water (NFW, Severn Biotech Ltd) and stored as 5 mg/mL stock solution at room temperature for further experiments.

c. Plasmid DNA amplification and extraction

The bacteria used to amplify the pDNA was modified with the plasmid (PLNT-SSFV-Luc-2A-eGFP) and was kindly provided by Professor Simon Waddington's group at the Institute for Women's Health, UCL. The plasmid DNA was isolated and purified from *E. coli* bacterial cells using the Invitrogen PureLink™ HiPure Plasmid Filter Maxiprep Kit (Catalogue No. K210016).

d. Details of transfection experiments

Cell preparation - HEK293T cells were grown in Dulbecco's modified Eagle's Medium (DMEM, Thermo Fisher Scientific) growth medium adjusted with 4.5 g/L D-glucose and GlutaMax™, supplemented with antibiotic-antimycotic solution: 10% (v/v) heat-deactivated fetal bovine serum (FBS) and 1% (v/v) penicillin/streptomycin (all products purchased from Gibco, Massachusetts, USA) at 37 °C and in a 5% CO_2 atmosphere.

Control experiments - The positive control was prepared according to the protocol for 12-well plate transfections[4], where 1.5 µL of the Lipofectamine 3000 transfection reagent was mixed with 50 µL of OptiMEM serum-free media (Gibco, Loughborough, UK) and vortexed for 2-3 s. In a separate Eppendorf tube, a mixture of 50 µL of OptiMEM with 1 µg pDNA and 2 µL of P3000 reagent was prepared. The two solutions were mixed by adding 50 µL of each (1:1 ratio), after which, the resultant 100 µL solution was incubated at room temperature for 10-15 min before adding the mixture to each well. The negative control was prepared by diluting 1 µg of pDNA up to 100 µL total volume with Opti-MEM, to ensure that the final volume added to the wells remained the same for all controls tested. The cells were transfected with a total pDNA content of 0.5, 1, 1.5, 2 and 2.5 µg/mL in each well. The quantity of pDNA added in each well was scaled in the solutions accordingly for varying pDNA content.

Nanogel formulations - Increasing amount of pDNA was added to the wells to determine the optimum amount necessary for transfection. Since we varied the pDNA content in the nanogel formulation, varying quantities of nanogel formulations were added to the wells to maintain constant the total pDNA delivered to the cells. **Table S1** gives information on these quantities of nanogel samples varied for each of the pDNA content delivered in the wells. These quantities were calculated using the dilution equation $C_i*V_i = C_f*V_f$; where C_i = pDNA concentration in the nanogel formulation (12.5 - 100 µg/mL), V_i = Volume of nanogel formulation added to each well (x µL), C_f = final pDNA content in the well (0.5 - 2.5 µg) and V_f = Total volume (1000 µL).

Table S1. Quantities of nanogel formulations used in transfection experiments.

pDNA concentration in nanogel formulation (µg/mL)	pDNA content in each well containing 1 mL growth media (µg)	Nanogel formulation added to each well (µL)
12.5	0.5	40
12.5	1	80
12.5	1.5	120
25	0.5	20
25	1	40
25	1.5	60
25	2	80
25	2.5	100
50	0.5	10
50	1	20
50	1.5	30
75	0.5	6.67
75	1	13.34
75	1.5	20
100	0.5	5
100	1	10
100	1.5	15

e. Quantitative assessment of GFP expression from fluorescence microscopy images

We used scikit-image (skimage)[5], an image processing toolbox in Python to quantitatively analyse the fluorescence microscopy images. To assess transfection efficiency from the images, the fluorescent blobs/cells that expressed GFP were detected via the toolbox in a custom python script. The images were first converted from the RGB format to grayscale format using the rgb2gray function of the skimage.color Python module. This allows to compute luminance (the perceived brightness of a colour) of an RGB image[6]. In a converted grayscale image, blobs are assumed to be light on dark background regions in the image. It is also assumed that the blobs are circular in shape. With these assumptions, the blobs were detected using the Laplacian of Gaussian (LoG) method[7] implemented in the blob_log function of the skimage.feature module. The blob_log function detects each blob in each grayscale image by finding the coordinates of the centre of the blob and the standard deviation of the Gaussian kernel that detected the blob. The radius of the detected blob is calculated as $\sqrt{2}\sigma$, where σ is the standard deviation of the Gaussian kernel that detected the blob. The parameters of the blob_log function were set at: min_sigma = 2, max_sigma = 30, num_sigma = 10 and threshold = 0.1.

The values of these parameters were determined using trial and error approach using several images. The number of blobs detected in each grayscale image can be directly obtained from the output of the blob_log function as this corresponds to the length of the 2d array returned by the function, with each row representing the coordinates of the blob and the standard deviation of the Gaussian kernel used to detect it. To improve the accuracy of these calculations, the number of blobs corresponding to the scale bar and text (legend in an image) were calculated separately and subtracted from the number of blobs and the total intensities respectively. Blob visualisations with overlaid circles were generated and saved as high-resolution images as shown in **Figure S2**. Quantitative results, i.e. the blob count were used for further statistical analysis. Note: Only the average number of fluorescent blobs/cells detected are reported in this study, as they provide a quantitative measure of the GFP expression.

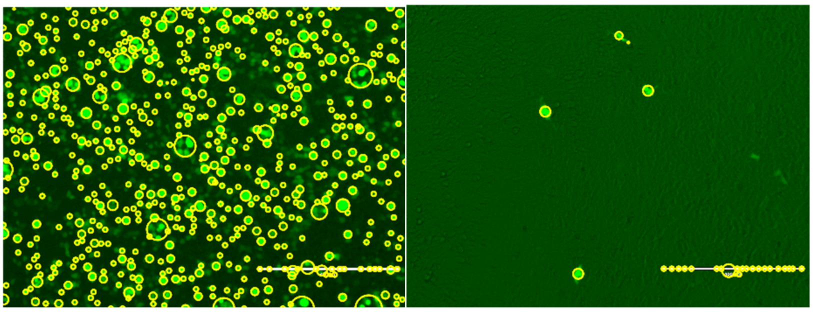

Figure S2. Detection of fluorescent cells (blobs) using the Laplacian of Gaussian method in custom Python script. The left image shows blob detection in a sample with many fluorescent cells (high density), while the right image shows detection in a sample with fewer fluorescent cells (low density).

Code availability - The Python code used to analyse microscopy images is openly available on GitHub and can be accessed at: https://github.com/arun-pn/blob-detector. Researchers using this code for data analysis are kindly requested to cite this publication.

f. Nanoparticle tracking analysis

Nanoparticle tracking analysis was performed to determine the yield of the synthesis by assessing the particle concentration in the formulations. Samples synthesised at different operating conditions, were investigated for particle concentration using the Nanosight LM10 instrument (Malvern Instruments, UK). The capture settings were: 532 nm green laser module, sCMOS camera type, three videos per sample, video length 30 s, camera level 7-9, slider shutter 86-890, slider gain 15. Captured videos were analysed using NanoSight 3.4 software, detection threshold of 5-7, auto settings for blur, minimum track length and jump mode. Samples were diluted in nuclease free water and measurements were carried out at room temperature in static mode.

S2. Optimisation of nanogel properties for gene delivery

Figure S3 displays transmission electron microscopy (TEM) images of nanogels produced at varying polymer concentrations and flowrate ratios used in the synthesis. Low particle concentration and a large free network for a high N/P ratio can be seen in **Figure S3 (d-e)** indicating the presence of free polymer in the formulation for these samples.

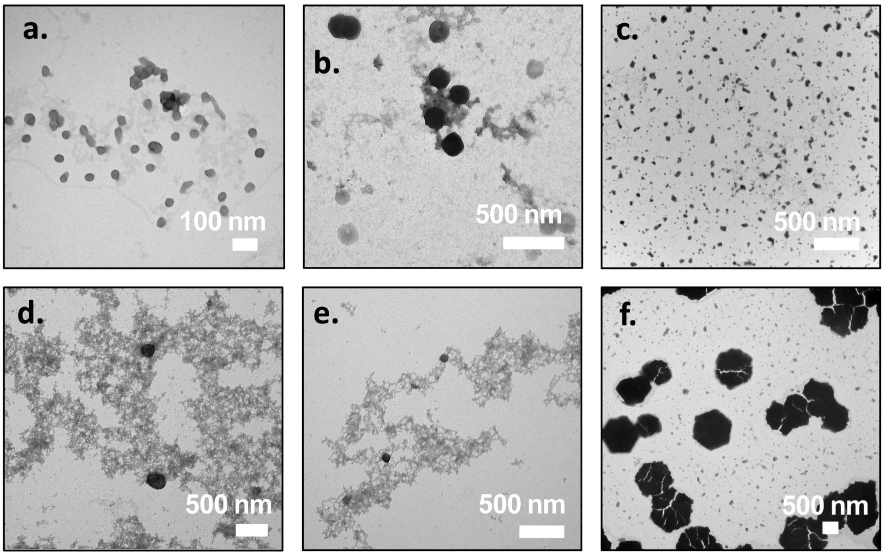

Figure S3. TEM images of nanogels synthesised at pDNA 25 µg/mL, TPP = 0.0095% W/V, RT = 31 s, and (a) N/P = 0.8, FRR = 0.1, (b) N/P = 3, FRR = 0.1, (c) N/P = 5, FRR = 0.1, (d) N/P = 30, FRR = 5, (e) N/P = 30, FRR = 0.1, feeds configuration PIS, and at higher TPP concentration for (f) N/P = 3, TPP 0.019% W/V, FRR = 0.1, PIS (pDNA = 25 µg/mL, RT = 31 s). Feeds configuration for all nanogels was PIC unless specified otherwise.

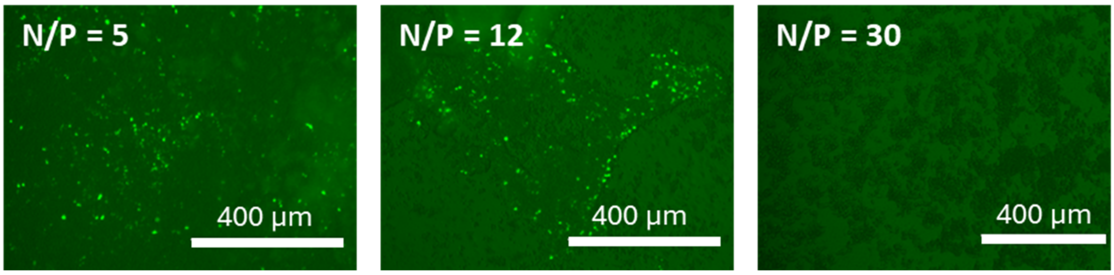

Figure S4. Fluorescence microscopy images of GFP expression in HEK293T cells at 72 h post-transfection for nanogels produced with high N/P ratios (as indicated). pDNA = 25 µg/mL, TPP = 0.0095 % W/V, FRR = 0.1 and RT = 31 s.

Increase in the polymer concentration led to a decrease in the transfection ability of the nanogels, as can be seen from very low to none GFP observed in **Figure S4**. Furthermore, the black blobs seen at N/P = 12 and 30 could imply that cell death had occurred, further suggesting low cell viability of the formulations synthesised at a high polymer concentration.

Figure S5 presents TEM images of nanogels synthesised during flow optimisation, highlighting morphological changes associated with varying TPP concentrations (0.5x – 10x) and pDNA loadings (12.5 – 100 µg/mL).

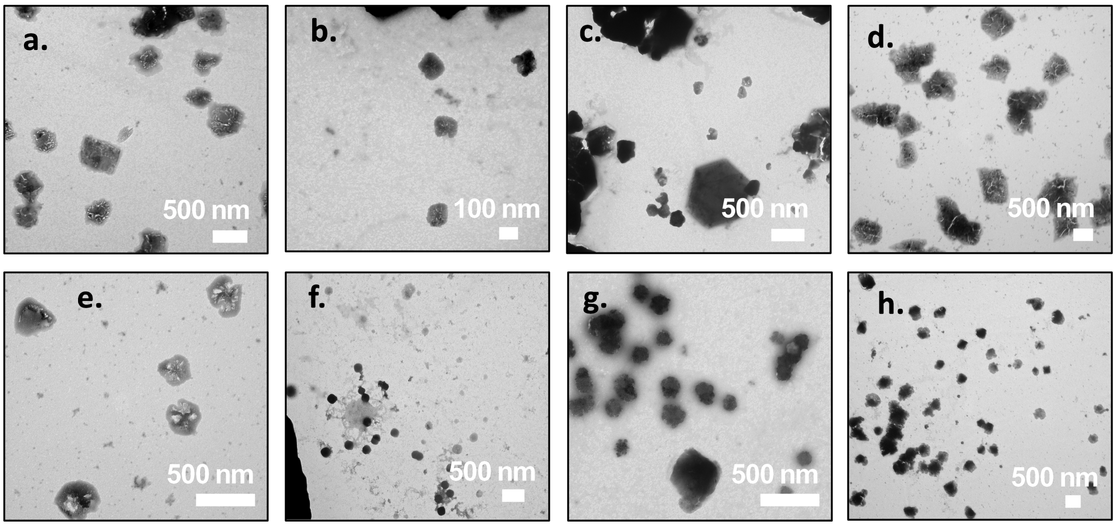

Figure S5. TEM images of nanogel synthesised at varying (a-d) TPP and (e-h) pDNA concentrations. Nanogels synthesised at N/P = 3, pDNA = 25 µg/mL, FRR = 0.1, RT = 31 s and varying TPP concentrations: (a) 0.0047% W/V, (b)-(c) 0.019% W/V, (d) 0.047% W/V. Nanogels synthesised at N/P = 3, FRR = 0.1, RT = 31 s and varying pDNA loadings: (e) 12.5 µg/mL, (f) 25 µg/mL, (g) 75 µg/mL and (h) 100 µg/mL. Detailed reagent concentrations are provided in Table 2 in the main paper.

Figure S6 demonstrates that the physicochemical properties of nanogels produced at a high polymer concentration and increasing residence time were of varying quality. The nanogel diameter and encapsulation efficiency increased with residence time, thus showing that short residence times were not sufficient for nanogel formation containing high polymer concentrations.

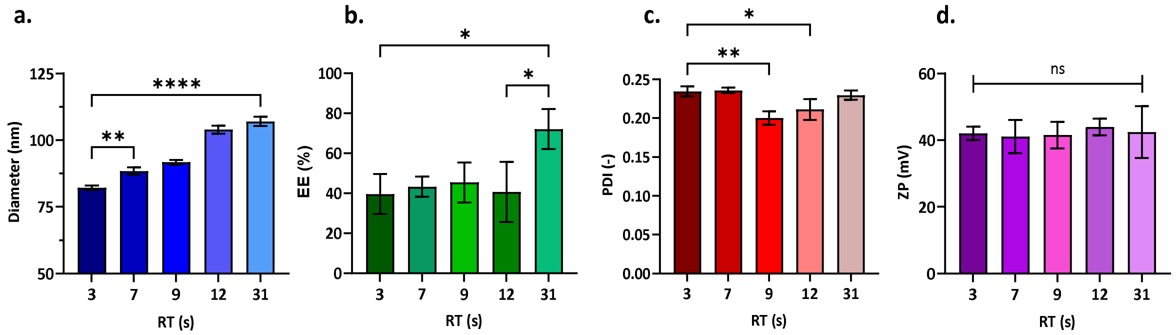

Figure S6. Physicochemical properties of nanogels produced at N/P = 12, FRR = 0.1 and varying residence time (RT), (a) nanogel size (diameter) vs RT; (b) Encapsulation efficiency, (EE) vs RT; (c) Polydispersity index, (PDI) vs. RT; and (d) Zeta potential, (ZP) vs. RT. Nanogels produced at pDNA = 25 µg/mL and TPP = 0.0095 % W/V.

S3. Hydrodynamic parameters in the coaxial flow reactor

The fluid velocities were calculated according to **equations S1** and **S2**, where Q_{core} and Q_{sheath} are the volumetric flowrates of the core and sheath stream respectively. The d_{core} and d_{sheath} represent the ID of core and sheath capillaries, while the D_{core} is the OD of the core capillary (0.6 mm). The average Reynolds number, Re_{avg} was determined using **Equation S4** using the kinematic viscosity of water (v). To calculate the Dean number, De for CFR V5, **Equation S5** was used, where r is the radius of curvature of the coil (50 mm). **Table S2** details the hydrodynamic parameters in the coaxial flow reactor experiments. All the experiments were performed with a velocity ratio, VR ≥ 1.

$$u_{core} = \frac{4Q_{core}}{\pi d_{core}^2} \quad \textbf{Equation S1}$$

$$u_{sheath} = \frac{4Q_{sheath}}{\pi\,(d_{sheath}^2 - D_{core}^2)} \quad \textbf{Equation S2}$$

$$u_{avg} = \frac{4(Q_{core} + Q_{sheath})}{\pi {d_{sheath}}^2} \quad \textbf{Equation S3}$$

$$Re_{avg} = \frac{d_{sheath}\, u_{avg}}{\nu} \quad \textbf{Equation S4}$$

$$De = Re_{avg}\sqrt{\frac{d_{sheath}}{2r}} \quad \textbf{Equation S5}$$

Table S2. Details of fluid velocities (u), Reynolds (Re) and Dean (De) numbers at varying volumetric flowrates (Q), residence time (RT) and flowrate ratios (FRR).

Reactor	**~RT**	Q_{core}	Q_{sheath}	u_{core}	u_{sheath}	u_{avg}	Re_{core}	Re_{sheath}	Re_{avg}	**FRR**	**Velocity ratio**	**De**
	**s**	**μL/min**	**μL/min**	**m/s**	**m/s**	**m/s**	**-**	**-**	**-**	**-**	u_{core}/u_{sheath}	**-**
CFR V3	3	176	1760	0.1906	0.0418	0.0328	29.85	42.06	41.05	0.1	4.56	-
	7	80	800	0.0866	0.0190	0.0149	13.57	19.12	18.66	0.1	4.56	-
	12	44	440	0.0476	0.0104	0.0082	7.46	10.51	10.26	0.1	4.56	-
	21	26	260	0.0281	0.0062	0.0048	4.41	6.21	6.06	0.1	4.56	-
	31	17.50	175	0.0189	0.0042	0.0033	2.97	4.18	4.08	0.1	4.56	-
CFR V3	31	4.72	188.8	0.0051	0.0045	0.0033	0.80	4.51	4.10	0.025	1.14	-
	31	9.22	184.3	0.0100	0.0044	0.0033	1.56	4.40	4.10	0.05	2.28	-
	31	25.25	168.3	0.0273	0.0040	0.0033	4.28	4.02	4.10	0.15	6.84	-
	31	44.67	148.8	0.0484	0.0035	0.0033	7.58	3.56	4.10	0.3	13.69	-
	31	55.30	138.2	0.0599	0.0033	0.0033	9.38	3.30	4.10	0.4	18.25	-
	31	64.52	129	0.0699	0.0031	0.0033	10.94	3.08	4.10	0.5	22.82	-
	31	160	32	0.1732	0.0008	0.0032	27.14	0.76	4.07	5	228.16	-
	31	175	17.5	0.1895	0.0004	0.0033	29.69	0.42	4.08	10	456.33	-
CFR V4	3	347.43	3474	0.3762	0.0824	0.0647	58.93	83.02	81.03	0.1	4.56	-
	7	160.19	1601	0.1734	0.0380	0.0298	27.17	38.28	37.36	0.1	4.56	-
	31	35.19	351.9	0.0381	0.0083	0.0065	5.97	8.41	8.21	0.1	4.56	-
CFR V5	3	1737.12	17371	1.8808	0.4122	0.3233	294.67	415.11	405.16	0.1	4.56	42.88
	7	800	8000	0.8661	0.1898	0.1489	135.70	191.17	186.59	0.1	4.56	19.75
	31	175.95	1759	0.1905	0.0417	0.0327	29.85	42.05	41.04	0.1	4.56	4.34

S4. Effect of pDNA amount, reagent feeds configuration, flowrate ratio on transfection experiments

Increasing amount of pDNA was added to the wells to determine the optimum amount of pDNA in the wells required to obtain a high GFP expression. **Figure S7** demonstrates the results from transfections performed with varying pDNA content in the wells for formulations prepared at different flowrate ratios and reagent feeds configurations. The transfection ability of nanogel formulations increases with the amount of pDNA added to the wells, as can be seen from the microscopy images. However, for samples produced with inverted streams (PIS feeds configuration) as well as at higher FRR = 10, no GFP was expressed even at higher pDNA content, thus indicating that poor (low-quality) formulations could not deliver even a small amount of genetic material to the cells.

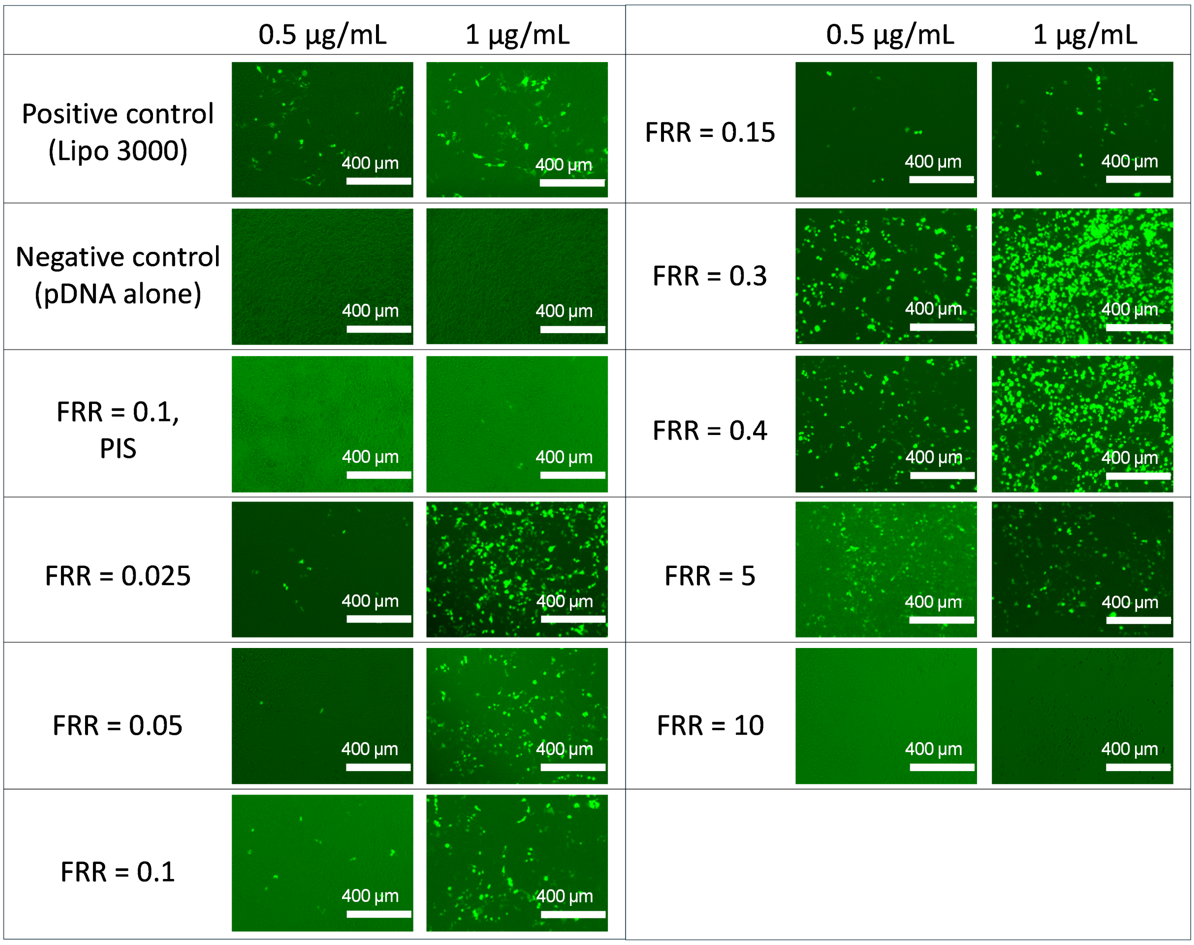

Figure S7. Fluorescence microscopy images of GFP expression in HEK293T cells at 72 h post-transfection shown for positive control (Lipofectamine 3000), negative control (pDNA without a vector) and nanogels synthesised with varying flowrate ratio (FRR). Columns indicate sample wells with a total pDNA concentration of 0.5 µg/mL and 1 µg/mL in the wells. The feeds configuration was PIC for all FRRs, unless mentioned otherwise. Nanogels were produced at N/P = 3, pDNA = 25 µg/mL, TPP = 0.0095 % W/V and RT= 31 s.

S5. Effect of reactor design and investigation of scalability

Scalability of the reactor was studied by increasing the reactor dimensions. The effect of reactor diameter and length on nanogel properties such as diameter, polydispersity index, encapsulation efficiency and zeta potential were assessed. **Figure S8** shows these properties.

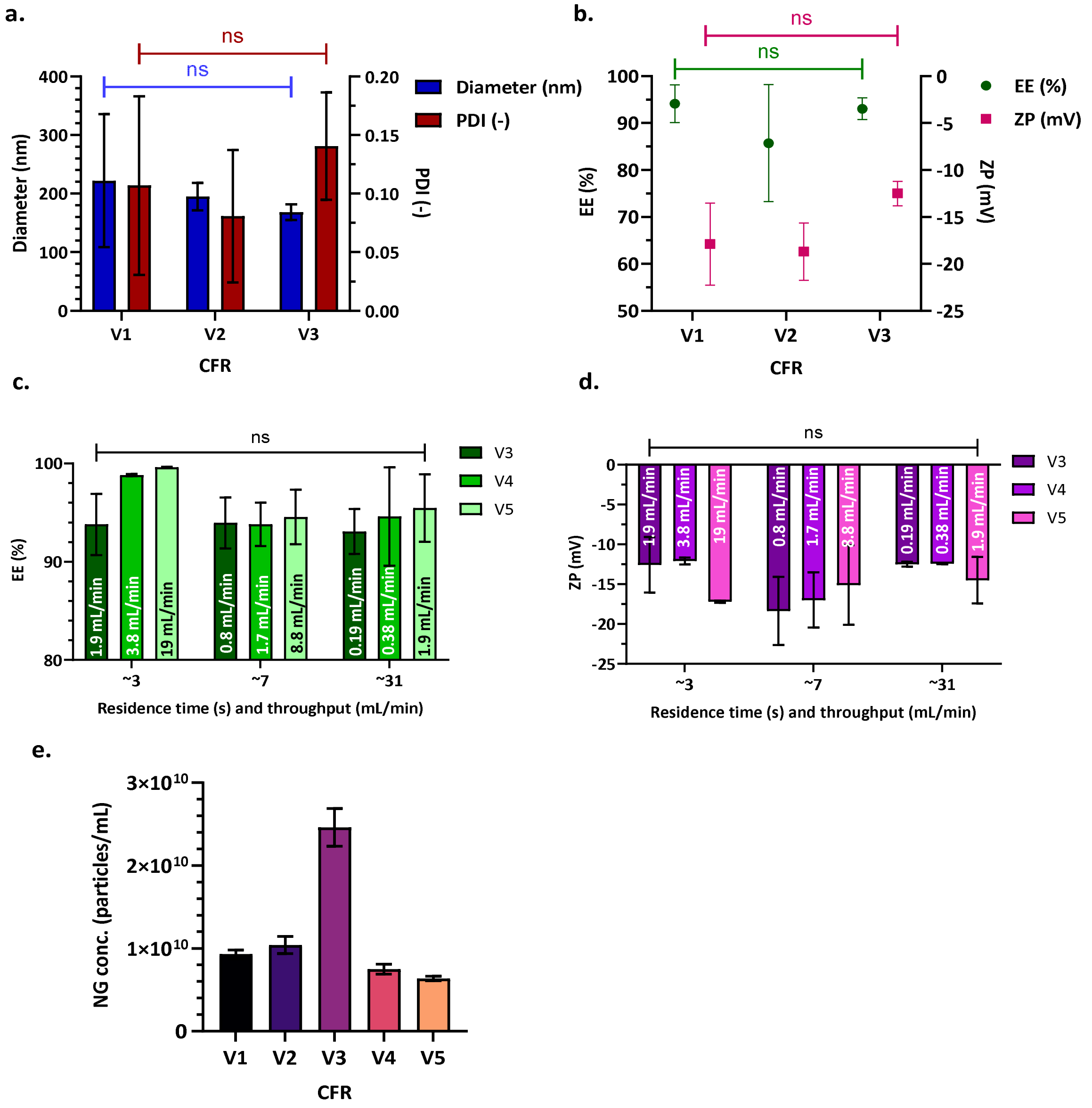

Figure S8. Assessment of scalability of the coaxial flow reactors (CFRs). (a) Nanogel size (diameter) and polydispersity index (PDI), for CFRs with different reactor diameters; (b) encapsulation efficiency, (EE) and zeta potential, (ZP), for CFRs with different reactor diameters; (c) EE vs. residence time (RT), for CFRs with different reactor lengths; (d) ZP vs. RT, for CFRs with different reactor lengths. Corresponding reactor throughputs are indicated within the bars. (e) Particle concentration in different CFR designs. For CFR dimensions, see Table 1. A one-way ANOVA followed by Tukey's post-hoc multiple comparisons test was performed to evaluate the effect of CFR diameter on nanogel diameter and PDI, for nanogels produced at RT 31 s. A two-way ANOVA followed by Tukey's multiple comparisons test was conducted to evaluate the effects of reactor length and throughput (RT) on the dependent variables, EE and ZP, separately. Plots show mean ± SD along with Tukey's HSD results. *P < 0.0486, **P < 0.0095, ***P < 0.0009, ****P < 0.0001. Nanogels were produced at N/P = 3, pDNA = 25 µg/mL, TPP = 0.0095 % W/V, FRR = 0.1, RT = 31 s for plots (a-b, e) and RT as specified in the plots for (c-d).

No significant changes in nanogel properties were observed with increasing reactor diameter, as confirmed by one-way ANOVA followed by Tukey's post-hoc multiple comparisons (**Figure S8 (a-b)**). With respect to increasing reactor length and throughput, a slight increase in nanogel diameter and a corresponding decrease in PDI were observed (**Figure 8 (a–c)**, main manuscript). However, no significant changes were noted in encapsulation efficiency or zeta potential **(Figure S8 (c–d))**, supported by a two-way ANOVA, indicating that reactor length and throughput had no significant effect on the encapsulation efficiency or zeta potential of the nanogels produced.

S6. Nanogel stability

A preliminary study was conducted to evaluate the stability of nanogel formulations. Nanogels synthesised under varying residence times and different reactors were stored at 4 °C and −18 °C for up to 48 h post-synthesis before being used for transfection. Stability was evaluated by comparing GFP expressions from samples transfected 48 h after storage (**Figure S9**) to those of fresh samples transfected within 12 h (**Figure 5, 8** – main paper). A marked decrease in the GFP expression was observed after 48 h of storage, with nanogels stored at −18 °C retaining slightly higher expression than those stored at 4°C, indicating modestly improved stability at lower temperatures. Fresh nanogels transfected within 12 h (stored at 4 °C) as shown in the main paper (**Figure 8 (d)**) demonstrated the highest GFP expression, suggesting stability is best maintained within this timeframe. While these results suggest a degree of stability under short-term storage conditions, further investigation - including physicochemical property changes over time, flow cytometry, stability at physiological pH - is required to comprehensively assess and enhance long term stability of these nanogels.

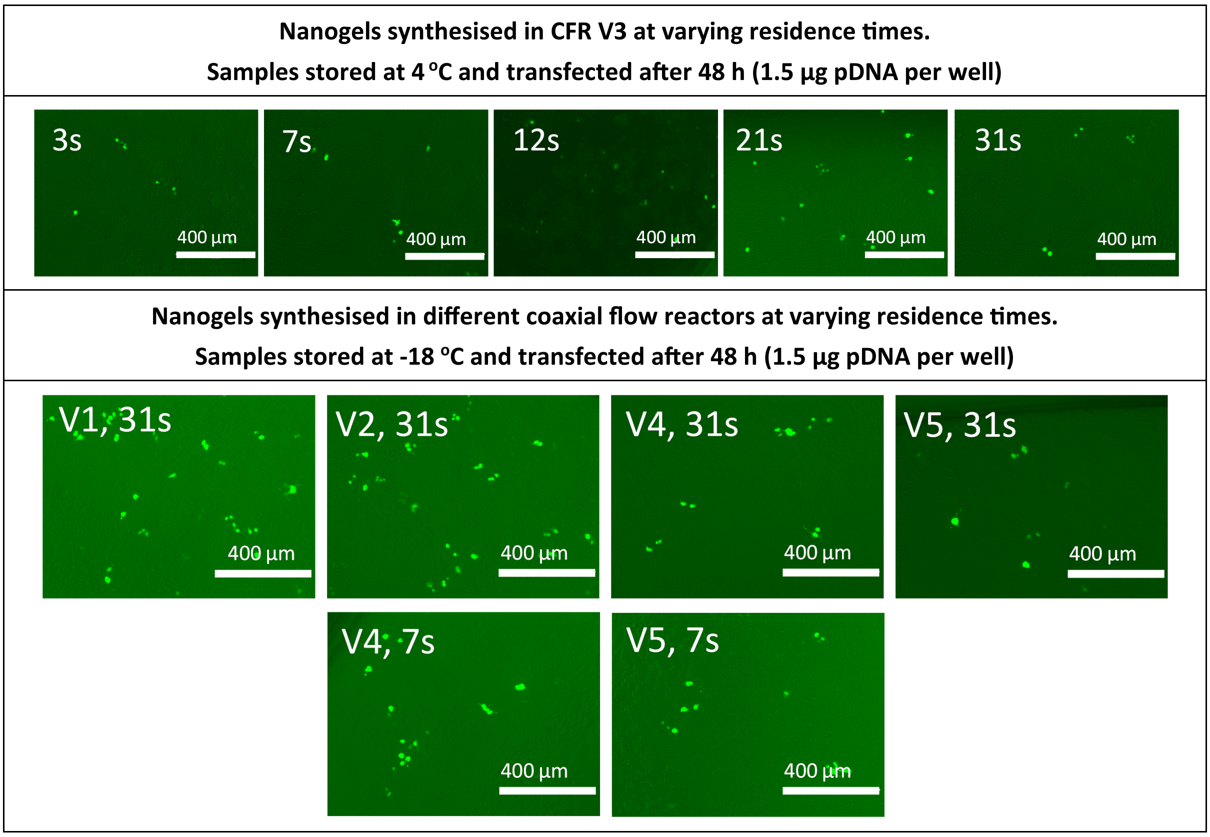

Figure S9. Fluorescence microscopy images of GFP expression in HEK293T cells at 72 h post-transfection nanogels synthesised in different coaxial flow reactors at varying residence times, with respect to sample storage temperature. Nanogels were produced with N/P = 3, pDNA = 25 µg/mL, TPP = 0.0095 % W/V, FRR = 0.1 and PIC feeds configuration.

References

1 S. C. Park, J. P. Nam, Y. M. Kim, J. H. Kim, J. W. Nah and M. K. Jang, *Int J Nanomedicine*, 2013, **8(1)**, 3663.

2 Z. Whiteley, G. Massaro, G. Gkogkos, A. Gavriilidis, S. N. Waddington, A. A. Rahim and D. Q. M. Craig, *Nanoscale*, 2023, **15**, 5865–5876.

3 S. Biswas, T. Ahmed, Md. M. Islam, Md. S. Islam and M. M. Rahman, in *Handbook of Chitin and Chitosan*, Elsevier, Amsterdam, 2020, vol. 3, ch. 14, pp. 433–470.

4 Invitrogen, Lipofectamine 3000 Reagent User Guide, https://assets.thermofisher.com/TFSAssets/LSG/manuals/lipofectamine3000_protocol.pdf, (accessed 16 July 2024).

5 S. van der Walt, J. L. Schönberger, J. Nunez-Iglesias, F. Boulogne, J. D. Warner, N. Yager, E. Gouillart and T. Yu, *PeerJ*, 2014, **2**, e453.

6 C. Poynton, 1997, preprint, https://poynton.ca/PDFs/ColorFAQ.pdf.

7 T. Lindeberg, *Int J Comput Vis*, 1993, **11**, 283–318.
